# Comparative Evaluation and Profiling of Chemical Tools for the Nuclear Hormone Receptor Family 2

**DOI:** 10.1021/acsptsci.4c00719

**Published:** 2025-02-20

**Authors:** Max Lewandowski, Romy Busch, Julian A. Marschner, Daniel Merk

**Affiliations:** 1 Ludwig-Maximilians-Universität München, Department of Pharmacy, 81377 Munich, Germany

**Keywords:** transcription factor, chemical tools, chemogenomics, orphan nuclear receptors, RXR

## Abstract

Nuclear receptors regulate transcription in response to ligand signals and enable the pharmacological control of gene expression. However, many nuclear receptors are still poorly explored and are not accessible to ligand-based target identification studies. In particular, most members of the NR2 family are among the least studied proteins of the class, and apart from the retinoid X receptors (RXR), validated NR2 ligands are very rare. Here, we gathered the NR2 modulators reported in literature for comparative profiling in uniform test systems. Most candidate compounds displayed insufficient on-target activity or selectivity to be used as chemical tools for NR2 receptors underscoring the urgent need for further NR2 ligand development. Nevertheless, a small NR2 modulator set could be assembled for application in a chemogenomic fashion.

There are 48 ligand-activated transcription factors in humans forming the superfamily of nuclear receptors (NRs, Figure 1a),^1,2^ which translate (endogenous) ligand signals into changes in gene expression patterns.^3^ The multifaceted roles of NRs span pivotal physiological processes, encompassing metabolism, inflammation, and cellular differentiation.^4^ Over decades, the NR1 and NR3 receptor families, including (steroid) hormone receptors and lipid sensors, have emerged as well-explored therapeutic targets of essential drugs like, for example, glucocorticoids as anti-inflammatory drugs, estrogen receptor modulators as contraceptives and anticancer agents, and PPAR agonists as oral antidiabetics.^5–7^ Despite this progress, a significant portion of the NR superfamily remains understudied, particularly within the NR2 family which comprises the hepatocyte nuclear factor-4 receptors (HNF4α/γ; NR2A1/2), the retinoid X receptors (RXRα/β/γ; NR2B1-3), the testicular receptors (TR2/4; NR2C1/2), the tailless-like receptors (TLX and PNR; NR2E1/3), and the COUP-TF-like receptors (COUP-TF1/2, V-erBA-related gene; NR2F1/2/6).^8,9^ Apart from RXR, all NR2 receptors are considered as orphan, and their ligands remain widely elusive. Therefore, chemical tools for most NR2 receptors are rare and poorly annotated posing an obstacle to target identification and validation studies. To enable chemogenomics on NR2 receptors and improve annotation, of the few available ligands, we gathered a scarce collection of NR2 modulators (agonists, antagonists, inverse agonists) for comparative evaluation and profiling. While the NR2B family (RXR) is well covered with high-quality ligands and a few early tools are available for NR2E1, we found the available ligands of the NR2A and NR2C families of insufficient quality to be used as chemical tools.

## Structure and Function of NR2 Receptors

**Figure 1:**
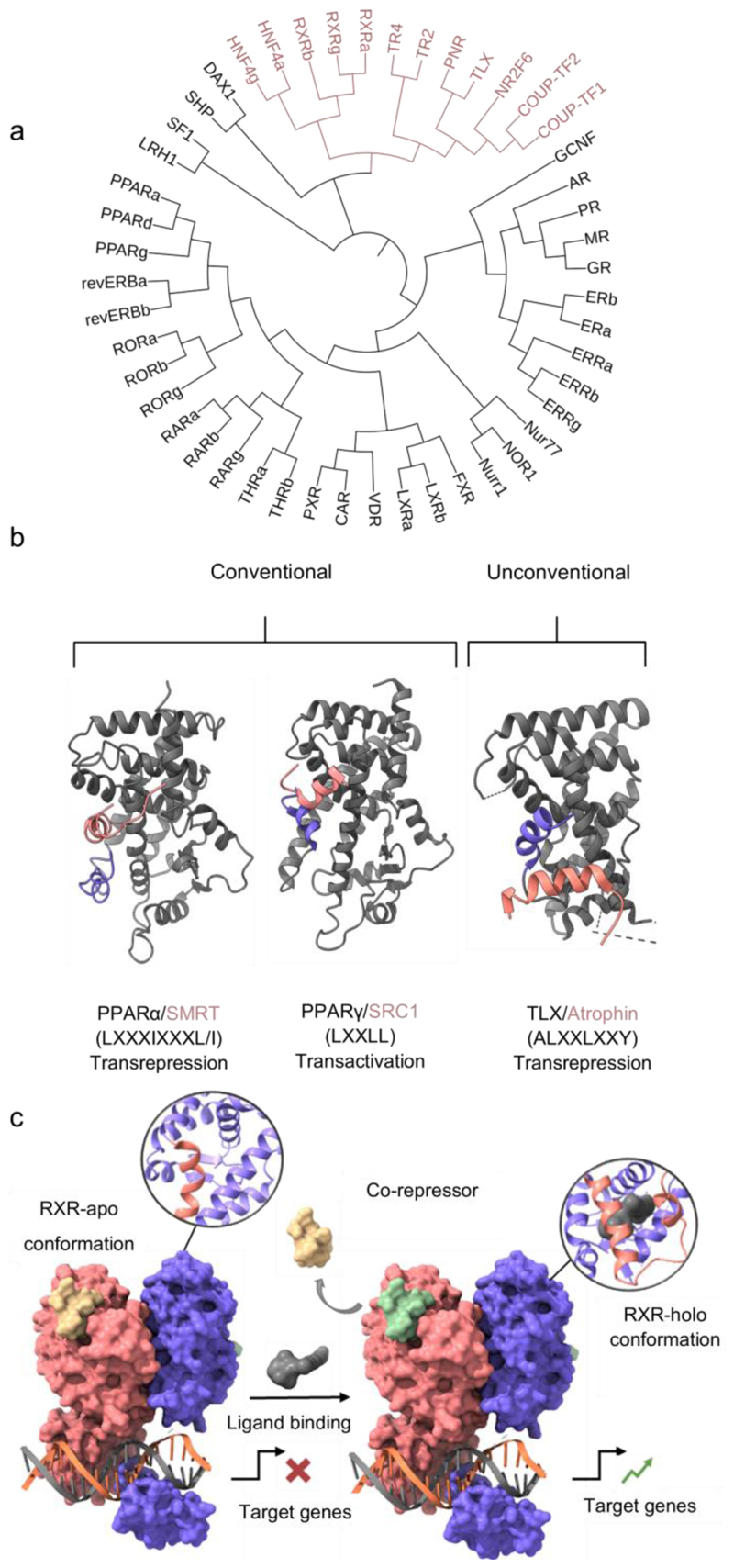
Structure and function of NR2 receptors. (a) Phylogenetic tree of the NR superfamily comprising 48 members in humans. The NR2 family is colored red. (b) Structural comparison of NRs. Conventional NRs (PPARγ as example) recruit corepressors (e.g., SMRT; pdb ID: 1KKQ^33^) in the ligand-free inactive state. Agonist binding relocates H12 (blue) to an active conformation occluding the corepressor binding site and forming a new surface for coactivator (e.g., SRC; pdb ID: 1FM6^34^) binding (schematic activation mechanism shown in (c)). Unconventional NRs acting as repressors of transcription (TLX with atrophin as example; pdb ID: 4xaj^31^) display a different conformation and position of the C-terminal helix which results in a binding site for atrophin-type corepressors beneath the terminal helix. (c) Schematic mechanism of (conventional) NR activation. Agonist binding induces corepressor release and promotes coactivator recruitment, to activate gene expression. (pdb ID: 5uan).

The archetypal structure of NRs correlates with their biological function and encompasses six common regions (Figure 1b). The N-terminal domain (NTD) is the least conserved among NRs and has a disordered and variable structure. As target of diverse post-translational modifications, and as site of the ligand-independent activation function 1 (AF-1), the NTD plays a role in regulating NR activity.^10–12^ The subsequent DNA binding domain (DBD) is the most conserved domain among NRs and is responsible for recognizing specific DNA sequences termed response elements (RE). The DBD spans approximately 70 amino acids^13^ and comprises two subdomains each with four cysteine residues coordinating a zinc ion in a zinc finger motif.^10^ The first subdomain features the DNA reading helix (helix 1), which interacts with the major groove and facilitates sequence-specific recognition of the hexameric half site of an RE.^3,10,14^ The second subdomain establishes non-specific interactions with the DNA backbone, and the adjacent loop - termed D-box - is important for receptor dimerization.^10,15,16^ The DBD is connected to the ligand binding domain (LBD) via a flexible hinge region which is also a target of post-translational modifications and contains a nuclear localization signal.^10,12,17^

The LBD mediates (endogenous) ligand binding and co-regulator interaction as key features for ligand-dependent activity (Figure 1c).^10,18,19^ Despite rather low conservation of the LBD over the NR family (<35%), it has a conserved architecture featuring 11-13 α-helices that form three antiparallel sheets, known as an α-helical sandwich.^20,21^ This intricate folding creates a hydrophobic cavity for the binding of lipophilic ligands in most NRs.^22,23^ Ligand binding via allosteric mechanisms induces conformational changes in the LBD to alter the NR’s activation state. The ligand-dependent activation function 2 (AF-2) located in the C-terminal helix of the LBD is highly dynamic and can form an interaction site for coregulator proteins containing an LXXLL motif.^10,20^ Ligand binding typically modulates the mobility of the C-terminal helix.^20,22,23^ While it is typically unordered and dynamic in the unliganded apo-state, agonist binding stabilizes the AF-2 in the activated conformation bound to the core of the LBD. This conformational change promotes the release of corepressors which are bound to the NR in the inactive state and the recruitment of coactivators (LXXLL motif) to form an activated holo-complex and initiate transcription.^20,22^ The dynamic properties of the C-terminal helix allow the existence of several states between the inactive and fully activated conformations. Therefore, different modulators may exhibit varying levels of activation efficacy, ranging from weak partial agonism to full agonism.^23–25^ Antagonists, by contrast, prevent the binding of (endogenous) agonists, without inducing conformational changes required for activation while inverse agonists stabilize the inactive state. The latter type of ligands is particularly relevant for NRs possessing an autoactivated conformation and thus constitutive activity like retinoic acid receptor related orphan receptors (ROR, NR1F) and NR4A receptors.^22,23,26^

In addition to ligand-dependent binding of coregulators, dimerization is critically involved in the regulation of NR activity. Although NRs can act as monomers on DNA, they more frequently adopt homodimeric structures or engage in heterodimeric complexes with RXR.^10,27^ This oligomerization adds another level of complexity and specificity as REs on the DNA distinguish different NR dimers.^10^ A large fraction of NRs mainly dimerize with RXR giving this member of the protein family particular importance.^1,10,27^ RXR heterodimers can be classified as permissive (PPAR/RXR, LXR/RXR, FXR/RXR), conditional (RAR/RXR) and nonpermissive (VDR/RXR, TR/RXR).^28^ In nonpermissive heterodimers, activity is independent of the presence of RXR agonists and RXR plays a subordinate role as a silent partner without directly influencing activity.^28,29^ In conditional and permissive heterodimers, in contrast, RXR ligands can affect activity and potentiate (conditional dimers) or even induce (permissive dimers) transcriptional activity.^28,30^ Therefore, RXR modulators can have manifold biological effects via the various RXR heterodimers and also relevant RXR homodimers.

Apart from the remarkable role of RXR as a universal dimer partner, the repressor activity of several NR2 receptors (NR2C and NR2E) is another important feature of this family. Diverging from conventional NR counterparts, these receptors share a common structural feature known as the autorepressed conformation.^31,32^ It is induced by a kink between helices 10 and 11 causing helix 11 to collapse into a space that typically corresponds to the ligand-binding site in the conventional NR. Consequently, the AF-2 obstructs the cofactor binding site, preventing the binding of common coactivators.^31,32^ This unconventional conformation, however, offers a binding pocket for a conserved Atro-box motif (ALXXLXXY) beneath helix 12 enabeling the recruitment of atrophin proteins, which may function as general corepressors for the repressive group of orphan nuclear receptors, as observed, for example, with TLX, PNR, TR4, and COUP-TF2. Disrupting this special structural feature through deletions or mutations in helix 12 has been shown to impair the ability of the receptors to repress gene transcription.^31^

### Roles and Potential of NR2 Receptors

Although the majority of the 12 NR2 receptors are among the least studied NR, there is preliminary evidence for the therapeutic potential of every NR2 member. Of note, apart from the widely distributed RXRs, most NR2 receptors display narrow expression patterns and are restricted to few tissues and organs, suggesting potential for specific therapeutic modulation with limited risk for systemic adverse effects. However, the lack of chemical tools to study the effects of pharmacological NR2 modulation as a therapeutic approach hinders further exploration of these promising targets.

The NR2A subfamily comprises the orphan hepatic nuclear factors HNF4α (NR2A1) and HNF4γ (NR2A2).^8^ HNF4α is predominantly expressed in hepatocytes, enterocytes, and pancreatic β-cells while its redundant paralog HNF4γ is exclusively found in the intestine.^35,36^ HNFs function as obligate homodimers^37^ with constitutive transactivation activity.^2^ HNF4α serves as a key regulator of genes involved in gluconeogenesis and lipid metabolism in liver, and acts as a master regulator of islet gene expression in the pancreas.^37–39^ Dysregulation of HNF4α is associated with gastrointestinal diseases, metabolic disorders, and gastrointestinal cancers.^38–41^ Most notably, mutations in the HNF4α gene cause the heritable form of type 2, diabetes maturity-onset diabetes of the young 1 (MODY-1), highlighting the receptor’s critical role in metabolic homeostasis.^42–44^

The NR2B subfamily comprises the three retinoid X receptor (RXR) subtypes, which exhibit a pivotal role in NR signaling as universal heterodimer partners. Due to their widespread distribution over all tissues and cell types, dysregulation of RXR signaling contributes to various pathologies such as metabolic and inflammatory diseases, cancer, and neurodegeneration.^4,45,46^ To date, bexarotene, the only FDA-approved RXR drug, has been extensively studied in clinical trials across various cancers, including non-small-cell lung cancer, acute myeloid leukemia, breast cancer, thyroid cancer, and melanoma. These trials consistently demonstrate therapeutic benefits, underscoring the potential of RXR modulation in malignancies.^47–50^ Beyond cancer, RXRs have been identified as promising targets for the treatment of neurodegenerative diseases, particularly multiple sclerosis (MS) and Alzheimer’s disease (AD).^51–57^

The orphan testicular receptors (TR2/4, NR2C) are involved in the transcriptional regulation of metabolism,^58,59^ bone physiology,^60^ and neuronal development.^61,62^ TR2 is primarily expressed in the prostate, seminal vesicle, and testis, while TR4 exhibits a broader expression profile across the testis, prostate, ovary, cerebellum, and hippocampus.^63,64^ Dysregulation in TR2 and TR4 activity has been associated with a spectrum of cancers (prostate, breast, liver, pituitary corticotroph tumors)^65–68^ and enhanced tumor metastasis and progression.^68^

The NR2E receptors’ tailless homologue (TLX, NR2E1) and photoreceptor-specific nuclear receptor (PNR, NR2E3) are orphan receptors with very specific expression profiles and a link to neuronal (TLX) and ocular (TLX, PNR) diseases. TLX is exclusively found in neuronal stem cells (NSCs) and retinal progenitor cells, and the only human NR with no documented hepatic expression.^69,70^ It is pivotal for maintaining NSCs in a proliferative and self-renewing state to enable neurogenesis.^71,72^

The characteristic autorepressed conformation of TLX allows binding of various corepressors, including atrophin1, histone deacetylases (HDACs), and lysine-specific demethylase (LSD1).^31,72,73^ Several lines of evidence support the potential of TLX in neurodegenerative diseases. TLX deficiency in rodents caused reduced neurogenesis, increased aggression, and cognitive deficits.^74,75^ Conversely, TLX overexpression enhanced memory function and learning.^72^ Additionally, involvement of TLX in psychiatric disorders (bipolar disorder and schizophrenia) as well as in various cancers has been proposed.^73,76,77^ PNR is found in retinal photoreceptor cells and plays a key role in both their development and maintenance.^32,78^ In the developing retina, PNR acts as a dual transcriptional modulator by repressing cone-specific genes and enhancing transcription of rod-specific genes, leading to the proper development of both rod and cone photoreceptors.^79,80^ PNR knockout resulted in disruption of the retinal architecture and a slow progressive retinal degeneration, characterized clinically by panretinal spotting, and histologically by whorls and rosettes.^81,82^ Furthermore, PNR is necessary for the repression of cone-specific transcription and the activation of rhodopsin expression in adult rod photoreceptor cells, ensuring the functional integrity of rods and preventing them from adopting a nonfunctional hybrid photoreceptor phenotype.^83^ Mutations in PNR gene are associated with several inherited retinopathies such as enhanced S-cone syndrome (Goldmann-Favre syndrome) and retinitis pigmentosa.^32,81,84–86^

The poorly studied NR2F family comprises the two closely related chicken ovalbumin upstream promoter transcription factors (COUP-TF1/2, NR2F1/2) as well as the more different NR2F6 receptor.^87^ COUP-TFs are expressed during early vertebrate development, where COUP-TF1 dominates in the peripheral and central nervous system and COUP-TF2 is present in the mesenchymal area of internal organs. COUP-TF1 is essential for central nervous system development^88^ while COUP-TF2 acts as a crucial regulator of cell differentiation and angiogenesis, contributing to tissue homeostasis and maintenance.^89–91^ In cancer, COUP-TFs have various roles, with COUP-TF2 affecting angiogenesis and tumor growth.^92^ NR2F6 is also involved in carcinogenesis and correlates with poor prognosis in several cancer types.^93–95^ Additionally, the NR2F receptors are involved in metabolic regulation and immune system modulation, particularly in T-cell differentiation and function.^96,97^

### Challenges in Orphan NR2 Receptor Ligand Discovery

Apart from the RXRs, which are the only well-characterized NR2 family members, identifying NR2 receptor modulators is challenging for several reasons. Most NR2 receptors have unique structural and functional features that hinder ligand discovery. The lack of high-quality cocrystal structures for many NR2 receptors restricts structure-based approaches and the absence of defined ligand binding sites, for example, in the transcriptional repressors NR2C and NR2E, where the classical orthosteric binding site is obstructed by a collapse of Helix 11, is a significant obstacle complicating molecular modeling and ligand design.^23,98^ Moreover, the pronounced hydrophobicity and broad specificity of ligand binding pockets, for example, in the NR2A family hinder the development of selective and high-affinity ligands.^23,99–101^ It has been proposed that (natural) ligands of certain receptors, such as the NR2A family, may act as structural components or prosthetic groups rather than as modulatory molecules, adding another layer of complexity to modulator design.^99,100^ Besides these structural challenges, incomplete knowledge on NR2 receptors provides obstacles in assay development. For example, poorly characterized coregulator interactions of many NR2 receptors hinder the use of coregulator recruitment assays based on fluorescence resonance energy transfer (FRET) or fluorescence polarization. Screening campaigns utilizing differential scanning fluorimetry (DSF) or cellular reporter gene assays have yielded promising ligands, but orthogonal validation is often inadequate as secondary assays are lacking.^101–106^

Nevertheless, progress has been made in ligand discovery for NR2 receptors, and a number of early chemical tool candidates have been reported in the literature as summarized in the following.

### Evaluation of NR2 Modulators as Chemical Tools

#### NR2A (HNF)

NR2A receptors have been deorphanized by the discovery of saturated fatty acids (e.g., palmitic and myristic acid) binding as natural ligands.^107^ However, fatty acids are not suitable as tools to study the biology of HNF4 and synthetic ligands are needed. The two HNF4 subtypes α and γ exhibit high similarity especially in the LBD and very similar ligand binding can hence be hypothesized. Despite the knowledge of natural ligands and the availability of cocrystal structures, HNF4 ligands are still very rare but a few potential chemical tools have been reported from three medium screening campaigns^108–110^ of drugs and drug fragments. Kiselyuk et al.^109^ discovered BIM5078 (**1**) as inhibitor of human insulin promoter activity and hypothesized HNF4 as molecular target. Quenching of the intrinsic Tyr/Trp fluorescence of HNF4α by **1** was considered as evidence of direct binding with an EC_50_ of 11.9 nM and BIM5078 (**1**) was found to suppress the expression of HNF4α and a set of its target genes in T6PNE cells. Using the same screening system, Lee et al.^108^ reported benfluorex (**2**) and alverine (**3**) as HNF4α agonists based on their ability to activate the human insulin promoter with intermediate micromolar EC_50_ values. Both drugs induced the expression of HNF4α and some HNF4α regulated genes, and diminished protease stability of HNF4α protein as a hint for direct interaction. A drug fragment screening using a Gal4-HNF4α hybrid reporter gene assay additionally identified the inverse HNF4α agonists **4** and **5**.^110^ Both compounds exhibited low micromolar potency in the reporter gene assay and binding affinity in ITC, and diminished expression of the HNF4 target gene fructose-1,6-bisphosphatase 1 (FBP1) in HepG2 cells. Nevertheless, the fragments **4** and **5** are too weak HNF4 ligands for use as tools and further optimization is required. The possibly more potent **1**-**3** have not been extensively validated or profiled for selectivity and nonspecific effects, and thus require additional evaluation and annotation before being considered as early tools to study HNF4 biology. We have hence acquired HNF4 modulators **1**-**3** as chemical tools and CG compound candidates for further characterization.

#### NR2B (RXR)

RXRs are the only NR2 subfamily that has been extensively studied in terms of ligand discovery and pharmacological modulation. Vitamin A metabolites^111^ and fatty acids^112^ are natural RXR activators, and several synthetic RXR agonist scaffolds have been designed based on the natural ligand 9-cis retinoic acid (reviewed in ^46^). The pan-RXR agonist bexarotene (**6**)^46,113–115^ is an approved drug for second-line cancer treatment and was used in various preclinical models to explore the potential of RXR in other diseases.^57,116^ However, **6** and related rexinoids are highly lipophilic^46,117^ and not fully selective^112,118^ for RXR. Accordingly, clinical use of **6** is associated with potentially severe adverse effects such as elevated triglyceride and cholesterol levels, leukopenia, hypothyroidism, and an increased risk of acute pancreatitis.^57,118^ Improved rexinoids such as V-125 and 9cUAB30, however, demonstrated that major drawbacks of bexarotene (hypothyreotism and elevated triglyceride levels) could be decoupled from RXR agonism.^119–122^ This has also inspired the development of further second-generation RXR agonists with improved features, selectivity, and safety/toxicity profiles.^24,123^ The RXR agonist **7** is available as a community approved chemical probe complying with highest quality criteria and offering a negative control compound (**8**).^123,124^ Medicinal chemistry efforts have additionally yielded several RXR antagonists (reviewed in refs ^46,125^) which were available via structural extension of agonist scaffolds. Furthermore, based on recent studies suggest that RXR side effects may result from overstimulation by common full agonists, several structurally diverse partial agonists^24,126^ have been developed, providing sufficient RXR activation for therapeutic effects while reducing adverse effects.^126–128^ As the three RXR subtypes (RXRα, RXRβ, RXRγ) exhibit remarkable similarity and comprise identical ligand pocket forming residues,^129,130^ the vast majority of RXR ligands exhibit equal activity on all three subtypes. Only very few RXR agonists with subtype preference^131–135^ have been identified so far, which indicates that selective modulation can be achieved, but significant efforts will be needed to eventually develop fully subtype selective RXR ligands. Valerenic acid^131^ (**9**) acting as RXRβ agonist (EC_50_ 5.2 µM, 69-fold act.) with strong functional preference over RXRα (EC_50_ 27 µM, 9-fold act.) and RXRγ (EC_50_ 43 µM, 4-fold act.) is a notable example and may have value as chemical tool for in vitro studies.

As various potent RXR agonists and antagonists are available as chemical tools for in vitro studies, the choice of an optimal orthogonal set should be made based on potency, selectivity, level of annotation, physicochemical properties, stability, absence of nonspecific cytotoxicity and availability to the community. Several RXR ligand scaffolds contain polyenic substructures (e.g., AGN-194204)^136^ and/or acrylic acid motifs (e.g., CD-3254)^137^ which both represent undesirable structural elements due to potential instability and PAINS character.

The chemical probe **7** is among the most potent and selective RXR agonists and suitable as a chemical tool in terms of activity profile and physicochemical features. Bexarotene (**6**) and the analogues LG100268 (**10**) and SR11237 (**11**) have been widely used as tools and are thus very broadly annotated. Despite having some off-targets, these RXR agonists add chemical orthogonality to **7** and thus appear as suitable candidates for an RXR targeting CG set. As the commercial availability of **10** is limited, **6** and **11** were selected for further profiling. The RXRβ preferential ligand **9** offers access to a preliminary evaluation of RXR subtype relevance. As RXR antagonists, UVI3003 (**12**) and HX531 (**13**)^138,139^ are potent, lack polyenic substructures and are commercially available to complement the set. **6, 7, 9**, and **11**-**13** were thus selected for comparative profiling to assemble an optimal set of chemical tools for RXR.

#### NR2C (TR)

NR2C ligands potentially suitable as chemical tools have not been reported in the literature to date. 44 putative NR2C modulators are annotated in ChEMBL (v. 33), but the majority of these reported activities are unrelated to NR2C and refer to TAK1. Only two compounds have been tested for NR2C modulation in the context of selectivity profiling of revERB modulators but were found inactive.^140^ The lack of NR2C ligands to study the biology of these orphan NR underscores the need for further efforts to develop chemical tools for unexplored NRs.^23^

#### NR2E (TLX, PNR)

Despite increasing interest in NR2E, ligands for this subfamily are still very rare and selective high-affinity modulators are lacking. In 2004, Benod et al. reported the discovery of a first set of TLX modulators from a differential scanning fluorimetry (DSF) based screening of approximately 20,000 compounds. 365 compounds inducing a Tm shift ≥0.9°C were considered as primary hits. Filtering for PAINs elements and privileged structural features predicted based on the TLX structure, secondary screening by surface plasmon resonance and full dose-response profiling resulted in three structurally unrelated TLX ligands (ccrp1-3, **14**-**16**) with K_d_ values of 6.6 μM, 650 nM, and 27.5 μM, respectively. Validation studies in a cellular reporter gene assay indicated TLX activation with EC_50_ values of 9.2 μM (**14**), 1.0 μM (**15**), and 250 nM (**16**) and **15** showed a trend toward SIRT and SLC1a1 but not p21 induction in human glioblastoma cells (T98G) in qRT-PCR experiments.^141^ Importantly, TLX expression was not affected by **15**.^103^ A small selectivity panel suggested a preference of **14-16** for TLX over ERβ, LXRβ (DSF), PNR, RXRα, and COUP-TF2 (reporter gene assays).^141^ Low toxicity in a WST-1 assay in HEK293T cells further supported the suitability of **15** as an early chemical tool to study TLX modulation despite low stability against microsomal degradation (*t*_1/2_ < 15 min). However, the TLX agonist activity of **15** was not reproducible by other groups^102,105^ and additional validation is required.

In 2020, the synthetic retinoic acid mimetic BMS453 (**17**) was reported as a TLX modulator in reporter gene assays (TLX/Gal4: IC_50_ 367 nM; full-length TLX: IC_50_ 159 nM). DSF and nuclear magnetic resonance (NMR) experiments suggested direct target engagement of **17** and a fluorescence polarization (FP) assay using a fluorescein isothiocyanate (FITC)-labeled atrophin nuclear receptor interaction fragment and unlabeled TLX LBD protein indicated displacement of atrophin by **17**. As a synthetic retinoid, **17** also exhibited agonism on RARs and RXRs.^142^ Additionally, **17** was found to exhibit pronounced toxicity in HEK293T cells used for the reporter gene assays.^103^

Screening of 480 drug fragments for TLX modulation in a reporter gene assay yielded 14 further TLX ligand scaffolds after orthogonal validation.^104^ Subsequently, propranolol (**18**) was identified as TLX agonist (Gal4-TLX: EC_50_ 32 µM; full-length TLX: EC_50_ 37 µM) with confirmed binding to the TLX LBD (K_d_ 0.5 µM in ITC). **18** also modulated TLX-regulated SIRT1 and PTEN/TET3 expression in qRT-PCR experiments. However, the β-adrenoceptor antagonist is too weak as a TLX modulator to be considered as a tool.^104^ Structural fusion of fragment screening hits additionally yielded the TLX agonist **19** (Gal4-TLX: EC_50_ 0.25 µM; full-length TLX: EC_50_ 0.7 µM) with high selectivity in the NR family. **19** also induced SIRT1 expression with no effect on TLX expression levels and ITC supported binding of **19** to the TLX LBD but revealed unfavorable thermodynamic properties.^102^

Evaluation of drug fragments also identified xanthines as TLX modulators with 1-methylxantine counteracting the repressor activity of TLX in reporter gene assays (IC_50_ 9 μM).^103^ Subsequent SAR studies revealed extension in 8-position with small (hetero-)aromatic systems and the alkylation pattern as driving factors for TLX activity.^103^ Comparison with the xanthine SAR for adenosine receptor antagonism highlighted 8-(2-chlorophenyl)-theophylline (**20**) as a TLX modulator (IC_50_ 0.5 µM; K_d_ 0.8 µM) with substantial preference over adenosine receptors.^103^ Direct interaction with the TLX LBD was evident from ITC and NMR-based binding studies. Moreover, the effects of xanthines on TLX activity were not affected by adenosine suggesting no involvement of the adenosine receptors and mutagenesis indicated interaction with an epitope around F226W/I230E in helix 5 of the TLX LBD. In mechanistic studies using labeled TLX LBD and homogeneous time-resolved FRET (HTRF), xanthines promoted TLX homodimerization and heterodimerization with RXR as potential molecular mode-of-action, and treatment of human glioblastoma cells with xanthines altered expression of the TLX-regulated genes SIRT1, p21 and SLC1a1. Despite the known activity of xanthine derivates on multiple targets and the lack of a fully TLX selective derivative, selected xanthines may contribute as early tools to studies on the biology of TLX.^103^

Overall, ligand discovery and optimization for TLX have yielded some chemical tool candidates that may be suitable to form an initial CG compound set. Considering commercial availability to enable broad use, we acquired **15, 17, 18** and the xanthine **21** for profiling as CG compound candidates. Additionally, we have included **19** in the profiling which is not commercially available but has been specifically designed as a TLX ligand.

Ligand discovery for PNR is also at a very early stage. Two high throughput screening (HTS) campaigns using either a cellular reporter gene assay based on Gal4-NCOR and PNR-VP16 fusion proteins (PubChem ID 602229)^143^ or an HTRF assay to observe the PNR-RetCOR interaction (PubChem ID 2300)^144^ each screened >300,000 compounds (Biomol Inc. Bioactive Lipid and Orphan Ligand libraries, or subset of the NIH library) for PNR modulation. A total of 2392 confirmed hits reducing reporter activity were obtained by the cellular approach^143^ and the HTRF assay campaign identified 379 primary hits^144^ but no selective PNR ligands and no follow-up structural optimization were reported. However, by further computer-aided evaluation of the cellular HTS results, Nakamura et al. identified two structurally unrelated synthetic inverse PNR agonists termed as photoregulin1 (**22**, PR1) and photoregulin3 (**23**, PR3).^143,145,146^ **22** (IC_50_ 0.25 µM) and **23** (IC_50_ 0.07 µM) repressed reporter activity in the Gal4-NCOR/PNR-VP16 assay and were inactive in control experiments using Gal4-VP16.^143^ In murine retinal cells, **22** and **23** caused a dose-dependent reduction of rhodopsin expression, which is a well-described target of PNR, in immunofluorescence and qRT-PCR experiments. **23** additionally decreased Rho mRNA expression.^145,146^ ITC data to confirm direct binding to PNR have only been reported for **23** (K_d_ 67 µM) but show high dilution heat and very weak (if any) heat of binding and a strong disconnect between cellular potency and affinity. Moreover, selectivity profiling of **22** and **23** is pending but essential to interpret their biological effects as also various other NR (particularly RXRs, THRβ, COUP-TFs, revERB, RORβ) are involved in photoreceptor cell development and maintenance.^147–150^

Connor et al.^151^ identified biliverdin (**25**) as an endogenous PNR ligand via MS-based binding experiments of retina extracts to PNR. Cellular reevaluation showed an approximately 3-fold PNR activation with an EC_50_ value of 5 nM in a reporter gene assay in 293F cells. A dose-dependent UV spectral change of biliverdin induced by the addition of MBP-PNR-LBD protein indicated direct binding with a K_d_ value of 200 nM. Interestingly, PNR mutations associated with Enhanced S-Cone Syndrome (ESCS) lowered the affinity of biliverdin to a K_d_ of 9 μM. Treatment of retinoblastoma cells with 1 µM biliverdin induced a two-fold increase in PNR protein levels according to Western blot analysis, which was considered as a hint for cellular target engagement as PNR has been reported to control its own expression.^151,152^ Selectivity testing by an MS-based binding experiment with MBP-NR2E1-LBD showed no interaction, indicating selectivity of biliverdin over TLX but effects on other NRs playing a role in the retina have not been evaluated.^151^

Overall, although very few PNR modulators have been reported to date, the inverse PNR agonists **22** and **23** emerge as early chemical tool candidates and the putative endogenous ligand biliverdin (**25**) was considered for profiling, too.

#### NR2F

The orphan NR2F receptors are poorly studied in terms of ligand discovery with only seven NR2F1 modulators and two NR2F6 modulators annotated in ChEMBL (v. 33). In 2021, Khalil et al. reported the first synthetic NR2F1 modulator from a virtual screening of 110,000 compounds on a model of the autorepressed NR2F1 LBD which was computationally transformed into an active conformation based on the LBD of RXRα. From 150 initial hits, 67 were selected for in vitro characterization in a reporter gene assay and C26 (**26**) was found to enhance reporter activity by approximately two-fold (EC_50_: 0.5-1 μM) in two cell lines. Cellular NR2F1 knockout experiments using CRISPR-Cas9 supported the NR2F1-dependence of the effects of **26**. Moreover, **26** induced expression of the NR2F1-regulated genes SOX9, RARβ, and p27 in chicken embryo chorioallantoic membrane tumors, and inhibited tumor growth and metastasis of head and neck squamous-cell carcinoma (HNSCC) in mice. However, **26** treatment of T-Hep3 cells inoculated onto chicken embryo chorioallantoic membrane caused a 2.3-fold increase in NR2F1 mRNA and a 10-fold increase in protein levels indicating potentially that induction of NR2F1 expression rather than direct activation mediated the observed effects.^153^

In 2020, Wang et al. reported inverse COUP-TF2 agonists from a reporter gene assay based cellular HTS of >300,000 compounds (Molecular Libraries Probe Production Centers Network chemical library; PubChem AID: 686940). Counter-screenings of primary hits using Gal4-VP16 supported the COUP-TF2 mediated activity of the structurally related inverse agonists CIA1 (**27**) and CIA2 (**28**) with IC_50_ values of 3.2 and 2.8 μM, respectively. Direct interaction with the receptor was investigated via a cellular thermal shift assay and in pulldown experiments using biotinylated **27**. Additionally, mutagenesis studies indicated a loss of binding of **27** to COUP-TF when W249 and F253 were mutated. Both **27** and **28** were profiled for selectivity on the related NRs HNF4α, RXRα, PPARα, LXRα, GR, PR, ER, RAR, RORγ, AR, and COUP-TF1 which revealed no relevant interaction in a pulldown assay. Based on the high structural similarity of NR2F receptors, it is surprising that no COUP-TF1 binding was observed. In prostate cancer cells, **27** and **28** treatment altered expression of the COUP-TF2 target genes FOXM1 and CDK1 suggesting cellular target engagement. Moreover, both compounds suppressed the growth of four prostate cancer cell lines with IC_50_ values ranging from 1.2 to 76 μM (**27**) and from 2.2 to 10.2 μM (**28**), respectively, and **27** (2.6 mg/kg) inhibited prostate cancer tumor growth and angiogenesis in a xenograft mouse model.^154^

Considering the very scarce collection of NR2F ligands, the only reported NR2F1 agonist **26** and the inverse NR2F2 agonist **27** appeared to be the best suitable as early chemical tool candidates for NR2 targeted CG and were included in further profiling (Table 1).^153,154^ **28** was excluded due to its close structural similarity to **27** and lack of commercial availability (Table 1).

### Comparative Profiling of NR2 Modulators

To probe the potential of the available NR2 ligands as chemical tools for CG, we evaluated their on-target activity and in-family selectivity in a uniform cellular Gal4-hybrid reporter gene assay platform.^158,159^ These assays rely on chimeric receptors composed of the LBD of a human nuclear receptor and the Gal4 DNA binding domain from yeast. A Gal4-responsive firefly luciferase construct served as reporter gene, and a constitutively expressed (SV40 promoter) renilla luciferase was used as internal control gene. This assay provides a favorably uniform setting to characterize nuclear receptor modulators and their selectivity. As a control experiment to observe unspecific effects on reporter transcription, we tested the effects of all compounds on the activity of the ligand-independent transcriptional activator Gal4-VP16^160^ replacing the chimeric nuclear receptor under otherwise identical conditions. As another key feature of chemical tools for cellular studies, we additionally tested all NR2 ligand tool candidates for their cytotoxicity in a multiplex toxicity assay monitoring proliferation, apoptosis, necrosis and metabolic activity using orthogonal reagents and dyes. The NR2 ligands were comparatively evaluated in these settings (Figure 3) at a minimal test concentration sufficient for full target engagement and chosen based on the observed cytotoxicity and reported activity profiles.

**Figure 2:**
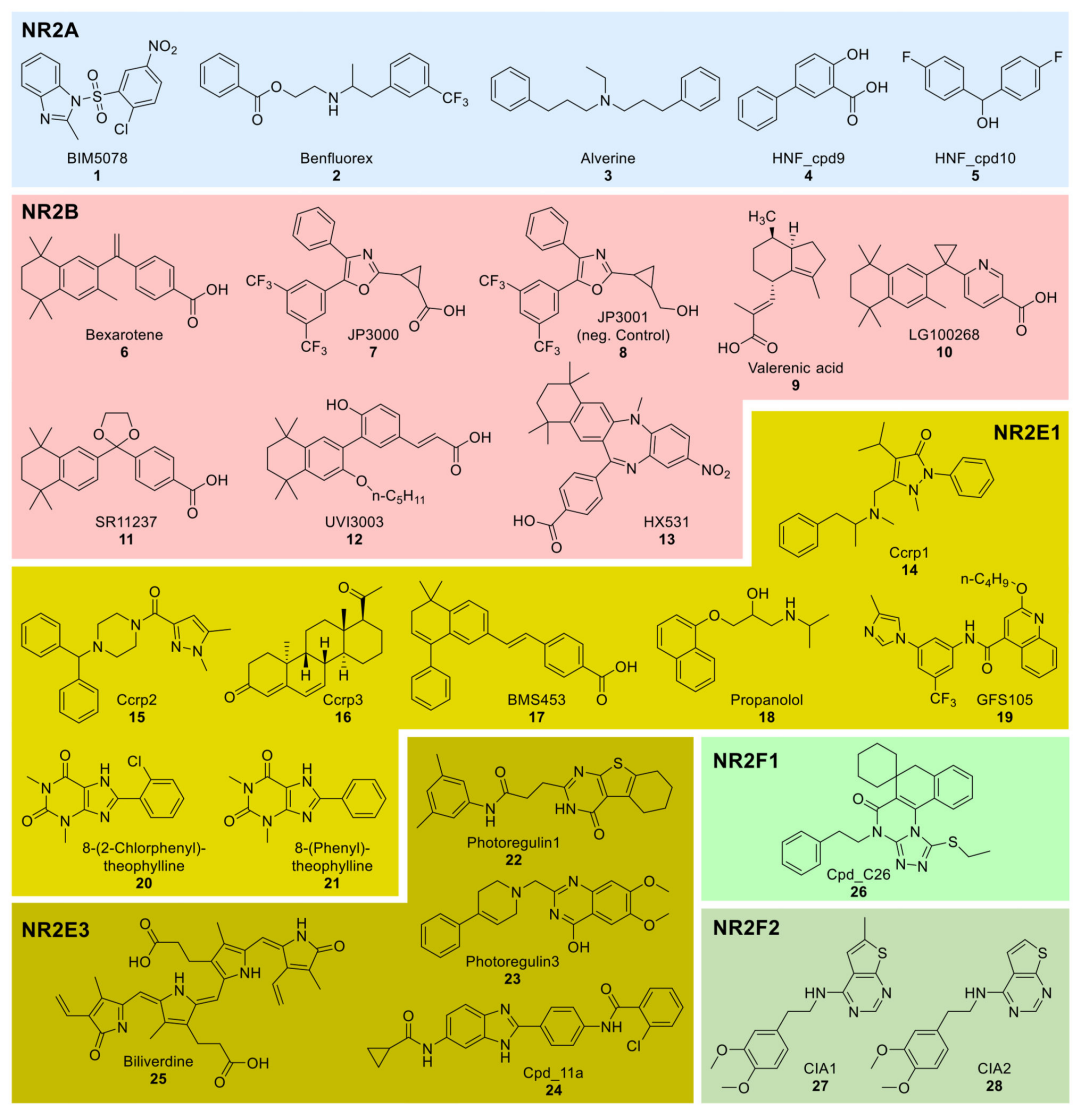
Chemical structures and common IDs of NR2 ligands reported in the literature. For RXR (NR2B), only a selected subset of highly potent examples is shown.

The NR2A (HNF4) ligand candidates **1**-**3** could not be validated in the hybrid reporter gene assay. Despite testing concentrations well above the reported EC_50_/IC_50_ values (Fig. 2), no effects on HNF4 activity were detectable for BIM5078 (**1**, IC_50_(lit.) 0.9 µM) at 3 µM, benfluorex (**2**, EC_50_(lit.) 24.4 µM) at 30 µM and alverine (**3**, EC_50_(lit.) 3.5-6.8 µM) at 30 µM. Despite lacking off-targets and toxic effects, **1**-**3** were thus disqualified as chemical tools or chemogenomics compounds for NR2A.

The hybrid reporter gene assay fully confirmed the activity of all of the studied NR2B (RXR) modulators. The full RXR agonists **6, 7** and **11** mediated strong RXR activation at 1 µM, the preferential RXRβ agonist valerenic acid (**9**) expectedly exhibited weak RXRα activation at 10 µM, and the RXR antagonists **12** and **13** robustly blocked bexarotene-induced RXR activation at 1 µM. At these active concentrations, the RXR modulators were nontoxic supporting suitability for cellular studies. The agonists **6** and **7** as well as the antagonists **12** and **13** were selective for RXR, whereas **6** also activated RAR, TR2, and PNR, and **11** displayed off-target activities on TR2, TR4, and PNR.

**Figure 3:**
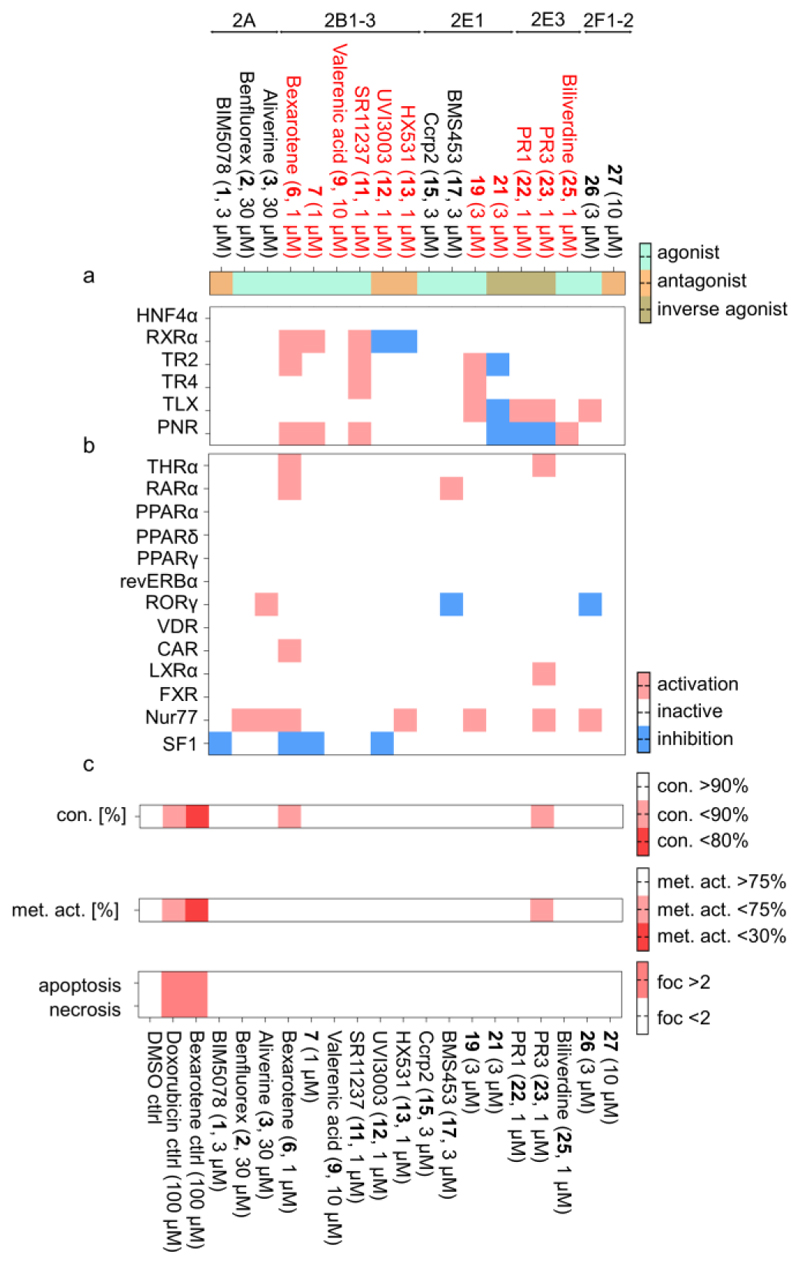
Comparative profiling of the NR2 modulators. Entities qualifying for a CG set are labeled red. (a) Effects of NR2 modulators on NR2 activity in uniform Gal4-hybrid reporter gene assays. Compounds were tested at concentrations around EC_90_ according to the literature or at the highest nontoxic concentration (from the multiplex toxicity assay). The heatmap shows the fold activation or repression of the respective NR2 receptors compared to 0.1% DMSO ctrl (for the transcriptional repressors TR2, TR4, TLX, and PNR, the reciprocal of ligand-induced repression is shown as fold activation for better comparison). Only effects are shown that are statistically significant against VP16 ctrl (Figure S2); *n* = 3. (b) Selectivity profiling of NR2 modulators in a representative panel of nuclear receptors outside the NR2 family. The heatmap shows the fold activation or repression of the respective NR2 receptors compared to 0.1% DMSO ctrl. Effects <5% of the respective reference ligands were considered as not relevant and omitted. Only effects are shown that are statistically significant against VP16 ctrl (Figure S3); *n* = 3. (c) Cytotoxicity profiling of NR2 modulators in a multiplex toxicity assay in COS-7 cells using orthogonal dyes and reagents to detect confluency, metabolic activity, apoptosis and necrosis. The heatmap shows relative compound effects compared to 0.1% DMSO ctrl; *n* = 4.

Profiling of putative NR2E1 (TLX) ligands confirmed TLX activation by **19** and inverse agonism by **21**. Both compounds were selective over other nuclear receptors and non-toxic at the selected active concentration and thus appear suitable as early tools. TLX modulation by **15** and **17** was not observed and, expectedly, **17** activated RAR. Modulation of NR2E3 (PNR) by **22, 23** as well as **26** was confirmed albeit with rather low efficacy. Favorable selectivity profiles and lack of cytotoxicity support **22, 23**, and **25** as early tools (Figure 3).

### Proof-of-Concept Application of Chemical Tools for NR2 Receptors in Functional and Phenotypic settings

**Figure 4:**
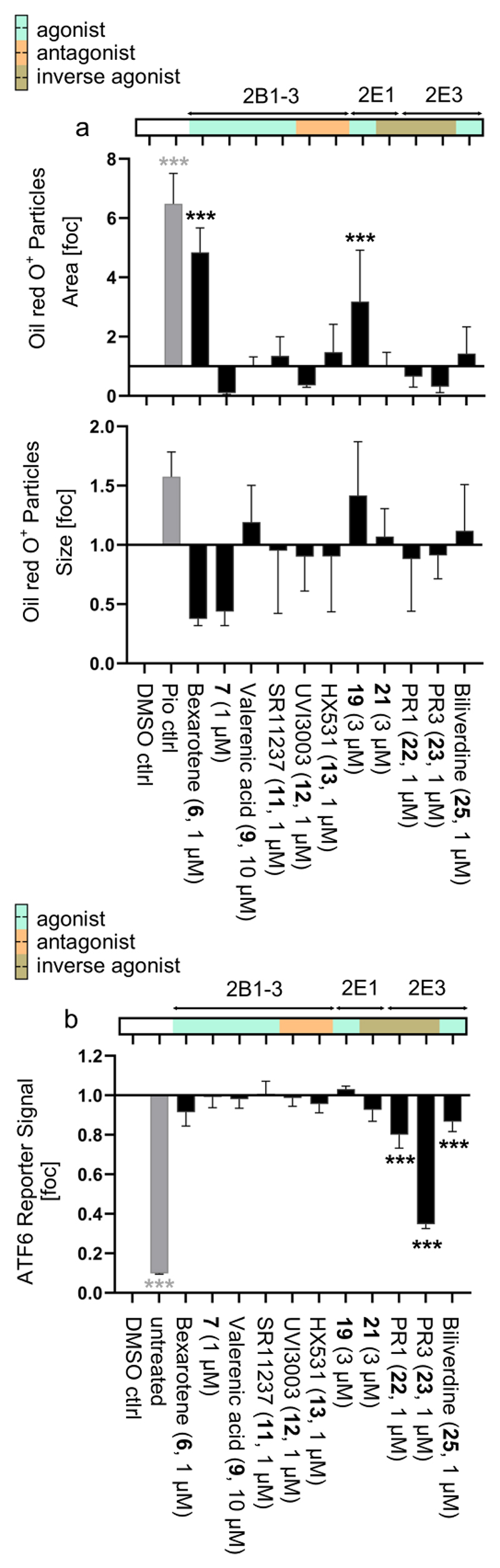
Application of validated NR2 modulators in phenotypic and functional assays in a chemogenomics approach.^161^ (a) Adipocyte-derived stem cell (ASC) differentiation with insulin, isobutylmethylxanthine, dexamethasone and the NR2 ligand collection. PPARγ agonist pioglitazone (1 µM) as positive control. Oil red O positive area (top panel) and droplet (particle) size are shown as the mean ± SD fold of DMSO ctrl; *n* = 4; *** p < 0.001 (ANOVA). (b) Effects of NR2 modulators on ATF6 activity in tunicamycin treated Hela ATF6 reporter cells. Data are the mean ± SD relative ATF6 reporter activity normalized to DMSO ctrl; *n* = 5; *** p < 0.001 (ANOVA).

Using the small collection of NR2 modulators with sufficient reproducible on-target activity and selectivity (**6, 7, 9, 11, 12, 13, 19, 21, 22, 23, 25**), we next explored their application as a chemogenomics set.^161^ To probe the potential of the collection to link phenotypic effects and target in a complex cellular setting, we determined compound effects on the differentiation of adipocyte-derived stem cells (ASC). Under cyclic treatment with insulin, isobutylmethylxanthine, dexamethasone and the PPARγ agonist pioglitazone,^162^ these cells develop into adipocytes and accumulate large amounts of fat. However, adipogenesis is orchestrated by many transcription factors^163^ and could potentially involve a role of NR2 receptors. Indeed, treatment of ASC with insulin, isobutylmethylxanthine, dexamethasone, and the NR2 ligand collection revealed diverse effects on differentiation and fat accumulation (Figure 4a). There was no uniform effect of RXR modulators on fat deposition in adipocytes, possibly due to diverse off-targets of **6** and **11**. The selective chemical probe **7** affected neither the overall Oil red O area nor the fat droplet (particle) size, indicating that RXR activation did not affect adipocyte differentiation. Among TLX ligands the agonist **19** increased the amount of fat and lipid particle size, pointing to enhanced adipogenesis. The inverse TLX agonist **21** had no effect. PNR ligand effects in the adipogenesis model were generally weak.

Next, we evaluated the potential impact of the NR2 modulator set on endoplasmic reticulum (ER) stress in an ATF6 activity assay (Fig. 4b). The activating transcription factor 6 (ATF6) participates in the unfolded protein response and is activated under ER stress.^164^ Tunicamycin-induced ATF6 activity was not affected by RXR and TLX modulators but was reduced by the inverse PNR agonists **22** and **23**. However, a consistent PNR effect could not be derived as the PNR agonist **25** slightly diminished ATF6 activity, too, suggesting that rather off-targets mediated the observed effects.

While these preliminary observations on the NR2 modulator impact on adipocyte differentiation and ER stress require further exploration, they indicate that the selected NR2 modulator set encompasses sufficient redundancy and chemical orthogonality to serve as a tool to generate new biological hypotheses in cellular settings.

## Conclusions

Several nuclear receptors of the NR1 and NR3 families mediate the effects of highly important and valuable drugs, demonstrating that members of this protein family generally hold strong therapeutic potential. Nevertheless, most NRs representing the NR2 family are still very poorly explored for their roles in health and disease, which is to a large part due to a lack of potent and well-characterized ligands. High-quality chemical tools are only available for RXR (NR2B) among the NR2 receptors, but even highly optimized RXR modulators lack subtype-selectivity, emphasizing the need for improved ligands for all NR2 receptors. Although a few synthetic modulators for other NR2 receptors have recently been reported, consistent in vitro characterization in uniform assays and independent validation are widely lacking. As a preliminary attempt to overcome this issue and identify potential early tools for the orphan NR2 receptors, we acquired NR2 ligands reported in the literature and subjected them to comparative profiling in a uniform assay platform to determine on-target activity and in-family selectivity in a cellular setting. This exercise failed to reproduce modulation of the respective NR2 target for several of the tested entities. Nevertheless, a few ligands for the NR2E family could be confirmed as early tools for in vitro studies, in addition to several well-characterized RXR modulators. Additionally, the 11 selected NR2 modulators displayed no cytotoxicity and sufficient NR selectivity at active concentrations for application in a chemogenomics fashion to probe the role of NR2B and NR2E receptors in phenotypic and functional cellular models.

The very scarce collection of NR2 ligands and the high attrition rate of compounds failing to modulate their annotated NR2 target in comparative profiling underscore that the coverage of this important protein family with chemical tools for target validation is insufficient. More attention of medicinal chemistry for NR2 modulators is urgently needed to provide high quality chemical tools for NR2 receptors to reveal new therapeutic opportunities.

### Experimental Procedures

#### Compound Selection

To identify modulators for NR2A, NR2C, NR2E and NR2F, data was extracted from “PubChem,^165^ ChEMBL (v.33),^166^ IUPHAR/BPS,^167^ chemicalprobes.org^168^ and Probes&Drugs (v. 02/2024).^169^ Additionally, Pubmed and google scholar were searched for scientific publications with the search terms “NR2A/NR2B/NR2C/NR2E/NR2F and modulator” or “HNF4/RXR/TR/TLX/PNR/COUP-TF and modulator”. NR2B ligands were rationally selected as described in the main text. Commercial availability for this resulting collection of NR2 ligand candidates was then checked (Sigma, Cayman Chemicals, Selleckchem, MilliporeSigma, BLD, and MedChem Express) and available compounds were acquired for comparative testing.

#### Adipogenesis Assay

Differentiation experiments of ASC52telo, hTERT cells (ATCC® SCRC-4000™) were conducted according to a previously described procedure.^162^ In brief, the cells were grown in DMEM high-glucose medium supplemented with 10% fetal calf serum, sodium pyruvate (1 mM), penicillin (100 U/mL), and streptomycin (100 μg/mL) at 37 °C and 5% CO_2_. The cells were seeded in standard culture medium at a density of 5x10^4^ cells per well in 96-well plates. After adherence overnight, cells were incubated with differentiation medium, composed of standard culture medium supplemented with human insulin (10 µg/mL, #I3536, Merck KgaA, Darmstadt, Germany), dexamethasone (1 µM, #D4902, Merck KgaA, Darmstadt, Germany,), isobutylmethylxanthine (0.5 mM, #I5879, Merck KgaA, Darmstadt, Germany), DMSO (final concentration 0.1%) and the respective test compounds or pioglitazone at 1 µM. The cells were kept in a differentiation medium for 48-72 h followed by a 24 h incubation with standard culture medium supplemented with 10 µg/mL insulin only. This cycle was repeated five times accounting for a total differentiation time of 18 days. The indicated test compounds at 0.1% DMSO or 0.1% DMSO alone as vehicle control were supplemented freshly with every medium exchange. After the 18-day differentiation procedure and test compound treatment, the cells were washed with phosphate buffered saline (PBS) once and fixed with formalin (10%, stabilized with methanol, 100 µL per well, #15071, Morphisto GmbH, Offenbach am Main, Germany) for 15 min at room temperature. The fixing solution was aspirated, and the fixed cells were washed twice with 50% ethanol solution with the second wash step incubating for 15 min at room temperature to equilibrate the specimens for staining. Oil Red O (#O0625, Merck KgaA, Darmstadt, Germany) was prepared at 0.5% (w/v) in 50% ethanol (v/v) and 5% salicylic acid (w/v), filtered with grade 595 Whatman® filter paper (#311611, Schleicher & Schuell GmbH, London, U.K.) and a 0.2 µm syringe filter (FP 30/0,2 CA-S, #10462200, Schleicher & Schuell GmbH, London, U.K.). Upon equilibration, specimens were incubated with 100 µL of 0.5% Oil Red O solution for 45 min at room temperature before the staining solution was aspirated and the wells were washed once with 50% (v/v) ethanol solution and three times with ddH_2_O. Specimens were kept in ddH_2_O for subsequent analysis. For each well, two pictures were taken at a 4x magnification using a Motic®AE31E inverted microscope and a Moticam 1080 (Motic Hong Kong Ltd.). Images were corrected for skewed illumination, Oil Red O crystal precipitates were identified and removed, and the resulting processed pictures were dichromized to extract gray values from the red channel using CellProfiler (Version 4.2.6). An appropriate greyvalue threshold was selected for the dichromized binary pictures and quantified (overall OilRed O positive area and average size of Oil Red O positive fat droplets, I.e. particles) using ImageJ 1.54f. Each sample was tested in four biologically independent experiments (*n* = 4).

#### Cytotoxicity Assay

COS-7 cells (DSMZ #ACC 60) were cultured at 37°C and 5% CO_2_ in DMEM high glucose medium supplemented with sodium pyruvate (1 mM), penicillin (100 U/mL), streptomycin (100 µg/mL), and 10% fetal calf serum (FCS). After aspirating the wells, the cells were seeded at 1x10^5^ cells per well in complete culture medium. After 24 h, the cells were treated with the respective test compounds (concentrations indicated in Figure 3), including positive controls Doxorubicin (100 µM) and Bexarotene (100 µM), in DMEM high-glucose medium, which was supplemented with 0.1% FCS, penicillin (100 U/mL), streptomycin (100 µg/mL) and 0.1% DMSO, or 0.1% DMSO alone as an untreated control. Each sample was prepared in at least 4 biologically independent replicates. After incubation for 24 h, the wells were aspirated and incubated for 30 min with stimulation medium containing 1 µM NucView® 405 fluorogenic caspase-3 substrate (#10405, Biotium, Fremont, USA) and Live-or-Dye Nuc-Fix Red (0.05x, Biotium, Inc., Fremont, CA, 1691 USA) to detect apoptosis and necrosis, respectively. After incubation, a total of 6 fluorescence images per well at 10x magnification were taken to detect NucView®-positive (Ex: 381–400 nm, Em: 414–450 nm) and Live-or-Dye-positive cells (Ex: 543−566 nm, Em: 580−611 nm) using on a Tecan Spark Cyto (Tecan Group AG). Reference readings for background correction and detection of auto-fluorescence were taken at 414–450 nm prior to staining. Thereafter, wells were aspirated, the cells were incubated for 3 h with 90 µL culture medium (0.2% FCS) and 10 µL Cell Counting Kit-8 solution (CCK-8, MedChem Express #HY-K0301), and absorbance was measured at 1, 2, and 3h of incubation at 450 nm on a Tecan Spark Cyto to assess metabolic activity of the cells. Before drug administration, after the first medium exchange, 24 h after drug administration, and after fluorescence imaging cell confluence was assessed using the Tecan Spark Cyto, to account for changes in cell confluence due to drug administration and cell handling. The number of apoptotic cells, metabolic activity, and changes in cell confluence in response to drug treatment were all normalized to the vehicle control of each biological replicate individually.

#### Hybrid Reporter Gene Assays

Gal4-hybrid reporter gene assays were performed as described previously using the plasmids pFA-CMV-hTHRα-LBD,^170^ pFA-CMV-hRARα-LBD,^171^ pFA-CMV-hPPARα-LBD,^172^ pFA-CMV-hPPARγ-LBD,^172^ pFA-CMV-hPPARδ-LBD,^172^ pFA-CMV-revERBα-LBD,^158^ pFA-CMV-hRORα-LBD,^173^ pFA-CMV-hLXRα-LBD,^118^ pFA-CMV-hFXR-LBD,^174^ pFA-CMV-hVDR-LBD,^171^ pFA-CMV-hCAR-LBD,^171^ pFA-CMV-hHNF4α-LBD,^110^ pFA-CMV-hRXRα-LBD,^175^ pFA-CMV-hTR2-LBD,^102^ pFA-CMV-hTR4-LBD,^102^ pFA-CMV-hTLX-LBD,^102^ pFA-CMV-hNR2E3-LBD,^102^ pFA-CMV-hNur77-LBD,^176^ pFA-CMV-hSF1-LBD^158^ encoding the hinge region and the LBD of the canonical isoform of the respective human nuclear receptors. pFR-Luc (Stratagene, La Jolla, CA, USA) was used as a reporter plasmid, and pRL-SV40 (Promega, Madison, WI, USA) was used for normalization of transfection efficiency and cell growth. HEK293T cells (German Collection of Microorganisms and Cell Culture GmbH, DSMZ) were cultured in Dulbecco’s modified Eagle’s medium (DMEM), high glucose supplemented with 10% fetal calf serum (FCS), sodium pyruvate (1 mM), penicillin (100 U/mL), and streptomycin (100 μg/mL) at 37 °C and 5% CO_2_ and seeded in 96-well plates (3 × 10^4^ cells/well). The medium was changed to Opti-MEM without supplements, and the cells were transiently transfected with one pFA-CMV-hNR-LBD clone, pFR-Luc, and pRL-SV40 using Lipofectamine LTX reagent (Invitrogen, Thermo Fisher Scientific) according to the manufacturer’s instructions. Five hours after transfection, cells were incubated with the test compounds in Opti-MEM supplemented with penicillin (100 U/mL), streptomycin (100 μg/mL), and 0.1% DMSO. Each sample was set up in duplicates and tested in at least three independent experiments. After 16 h incubation, luciferase activity was measured using the Dual-Glo Luciferase Assay System (Promega) according to the manufacturer’s protocol on a Tecan Spark luminometer (Tecan Deutschland GmbH, Crailsheim, Germany). Firefly luminescence was divided by Renilla luminescence and multiplied by 1000 resulting in relative light units (RLUs) to normalize for transfection efficiency and cell growth. Fold activation was obtained by dividing the mean RLU of the test compound by the mean^a^ RLU of the untreated control, and relative activation was calculated by dividing the fold activation of a test sample by the fold activation of the respective reference agonist. Antagonistic effects were evaluated by coincubation of the test compound with the respective reference ligand. The following reference ligands were used: THRα–1 μM T3; RARα–1 μM tretinoin; PPARα–1 μM GW7647; PPARγ–1 μM pioglitazone; PPARδ–1 μM L165,041; RORγ–1 μM SR1001; LXRα–1 μM T0901317; FXR–1 μM GW4064; VDR–1 μM calcitriol; CAR–1 μM CITCO, RXRα–1 μM bexarotene.

#### ATF6 Activity Assay

Hela cells containing a Renilla luciferase reporter gene for ATF6 activity (ATF6 Leeporter™ Luciferase Reporter-HeLa Cell Line, Abeomics Inc.; #14-138ACL) were cultured in DMEM supplemented with 10% heat-inactivated FCS, penicillin (100 U/mL), streptomycin (100 μg/mL) and puromycin (3 µg/mL) at 37°C and 5% (v/v) CO_2_ and seeded in 96-well plates (1 × 10^5^ per well). After 24 h, the cells were incubated with the test compounds solubilized with 0.1% DMSO in the same medium without puromycin. After 4 h of incubation, tunicamycin (100 ng/mL) was added. After another 18 h of incubation, cells were assayed for renilla luminescence using DualGlo reagent (Promega) on a Tecan SPARK Cyto (Tecan). Test samples were normalized to the 0.1% DMSO control.

## Supplementary Material

Supporting info.

## Figures and Tables

**Table 1:** NR2 Ligands with Their Main NR2 Targets, Activity Type, Reported Cellular Activity (Reporter Gene assay^a^, Cell Differentiation assay^b^, Insuline Promotor assay^c^), Biophysical Evidence of Binding (ITC, SPA, FP/HTRF-Based Recruitment Assay, or Cocrystal Structure), Reference and Commercial Availability.

Structure ID	Target	Type	Reference	Biological activity/potency		CAS	Commercial availability
				Cellular	Biophysical		
BIM5078 (**1**)		Antagonist	10.1016/j.chembiol.2012.05.014^109^	IC_50_: 0.93 μM ^c^	Kd: 0.012 μM ^e^	337506-43-1	Yes
Benfluorex (**2**)	NR2A	Agonist	10.1021/cb4000986^108^	EC_50_: 24.4 μM ^c^	n.a.	23642-66-2	Yes
Alverine citrate (**3**)		Agonist		EC_50_: 3.5-6.8 μM ^c^	n.a.	5560-59-8	Yes
HNF_cpd9 (**4**)		Inverse Agonist	10.3390/ijms21217895^110^	IC_50_: 8.0 μM ^a^	K_d_: 0.30 μM ^f^	323-87-5	Yes
HNF_cpd10 (**5**)		Inverse Agonist		IC_50_: 24 μM ^a^	K_d_: 1.70 μM ^f^	365-24-2	Yes
Bexarotene (**6**)	NR2B1-3	Agonist	10.1021/jm00044a014^115^	RXRα: 0.033 μM ^a^ RXRβ: 0.024 μM ^a^ RXRγ: 0.025 μM ^a^	RXRα: 0.014 μM ^g^ RXRβ: 0.021 μM ^g^ RXRγ: 0.029 μM ^g^	153559-49-0	Yes
JP3000 (**7**)		Agonist	10.1021/acs.jmedchem.3c01435^123^	RXRα: 0.005 μM ^a^ RXRβ: 0.0014 μM ^a^ RXRγ: 0.004 μM ^a^	<0.001 μM ^g^	3034097-74-7	Yes
Valerenic Acid (**9**)		Agonist	10.1021/acs.jmedchem.8b00494^131^	RXRα: 27 μM ^a^ RXRβ: 5.2 μM ^a^ RXRγ: 43 μM ^a^	n.a.	3569-10-6	Yes
LG100268 (**10**)		Agonist	10.1021/jm00016a018^115^	RXRα: 0.004 μM ^a^ RXRβ: 0.003 μM ^a^ RXRγ: 0.004 μM ^a^	RXRα: 0.003 μM ^g^ RXRβ: 0.003 μM ^g^ RXRγ: 0.003 μM ^g^	153559-76-3	Yes
SR11237 (**11**)		Agonist	10.1002/cmdc.200700265^135^	RXRα: 0.029 μM ^a^ RXRβ: 0.098 μM ^a^ RXRγ: 0.232 μM ^a^	n.a. co-crystal: 2zy0	146670-40-8	Yes
UVI3003 (**12**)		Antagonist	10.1073/pnas.0705356104^138^	RXRb: ~0.050 μM ^a,j^	n.a.	847239-17-2	Yes
HX531 (**13**)		Antagonist	10.1248/cpb.47.1778^139^	IC_50_: 0.018 μM ^b^	n.a.	188844-34-0	Yes
Ccrp2 (**15**)	NR2E1	Agonist	10.1371/journal.pone.0099440^155^	EC_50_: 1.0 μM ^a^	K_d_: 0.65 μM ^d^	958323-31-4	Yes
BMS453 (**17**)		Agonist	10.1016/j.chembiol.2020.07.013^156^	IC_50_: 0.367 μM ^a^ IC_50_: 0.159 μM ^a^	K_d_: ~1.0 μM ^i,j^	166977-43-1	Yes
Propranolol (**18**)		Agonist	10.1021/acs.jmedchem.1c00733^104^	EC_50_: 32 μM ^a^	K_d_: 0.50 μM ^f^	318-98-9	Yes
GFS105 (**19**)		Agonist	10.1021/acs.jmedchem.1c01757^102^	IC_50_: 0.25 μM ^a^ EC_50_: 0.7 μM ^a^	K_d_: 0.50 μM ^f^	n.a.	No
8-Phenyl- theophylline (**21**)		Inverse Agonist	10.1021/acsptsci.1c00195^103^	IC_50_: 0.5 μM ^a^	K_d_: 0.80 μM ^f^	961-45-5	Yes
Photoregulin1 (**22**)	NR2E3	Inverse Agonist	10.1167/iovs.16-20177^146^	IC_50_: 0.25 μM ^a^	n.a.	1003736-23-9	Yes
Photoregulin3 (**23**)		Inverse Agonist	10.7554/eLife.30577^145^	IC_50_: 0.070 μM ^a^	K_d_: 64 μM ^f^	785708-33-0	Yes
Compound11a (**24**)		Agonist	10.1016/j.bmcl.2006.07.056^157^	EC_50_: 0.141 μM ^a^ EC_50_: 0.035 μM ^a^	n.a.	911211-69-3	No
Biliverdin (**25**)		Agonist	10.1038/s41598-022-11502-3^151^	EC_50_: 0.005 μM ^a^	Kd: 0.20 μM ^h^	856699-18-8	Yes
Cpd_C26 (**26**)	NR2F1	Agonist	10.1084/jem.20210836^153^	EC_50_: n.a.; 1.9 f.A. @ 1 μM ^a^ EC_50_: n.a.; 2.4 f.A. @ 0.1 μM ^a^	n.a.	374101-64-1	Yes
CIA1 (**27**)	NR2F2	Antagonist	10.1126/sciadv.aaz8031^154^	IC_50_: 3.2 μM ^a^	n.a.	1049691-47-5	Yes
CIA2 (**28**)		Antagonist		IC_50_: 2.8 μM ^a^		n.a.	No

a From reporter gene assay.

b From cell differentiation assay.

c From insulin promoter assay.

d K_d_ value from DSF assays.

e K_d_ value from Try/Trp fluorescence quenching assays.

f K_d_ value from ITC.

g K_d_ value from radioligand binding studies.

h K_d_ value determined by UV absorption.

i K_d_ value from FP assay.

j No precise value reported.

## References

[1] Germain P, Staels B, Dacquet C, Spedding M, Laudet V (2006). Overview of Nomenclature of Nuclear Receptors. Pharmacol Rev.

[2] Alexander SP, Cidlowski JA, Kelly E, Mathie A, Peters JA, Veale EL, Armstrong JF, Faccenda E, Harding SD, Pawson AJ, Southan C (2021). THE CONCISE GUIDE TO PHARMACOLOGY 2021/22: Nuclear Hormone Receptors. Br J Pharmacol.

[3] Aranda A, Pascual A (2001). Nuclear Hormone Receptors and Gene Expression. Physiol Rev.

[4] Evans RM, Mangelsdorf DJ (2014). Nuclear Receptors, RXR, and the Big Bang. Cell.

[5] Moore DD, Kato S, Xie W, Mangelsdorf DJ, Schmidt DR, Xiao R, Kliewer SA (2006). International Union of Pharmacology. LXII. The NR1H and NR1I Receptors: Constitutive Androstane Receptor, Pregnene X Receptor, Farnesoid X Receptor α, Farnesoid X Receptor β, Liver X Receptor α, Liver X Receptor β, and Vitamin D Receptor. Pharmacol Rev.

[6] Rhen T, Cidlowski JA (2005). Antiinflammatory Action of Glucocorticoids-New Mechanisms for Old Drugs. N Engl J Med.

[7] Patel HK, Bihani T (2018). Selective Estrogen Receptor Modulators (SERMs) and Selective Estrogen Receptor Degraders (SERDs) in Cancer Treatment. Pharmacol Ther.

[8] Benoit G, Cooney A, Giguere V, Ingraham H, Lazar M, Muscat G, Perlmann T, Renaud JP, Schwabe J, Sladek F, Tsai MJ (2006). International Union of Pharmacology. LXVI. Orphan Nuclear Receptors. Pharmacol Rev.

[9] Germain P, Chambon P, Eichele G, Evans RM, Lazar MA, Leid M, De Lera AR, Lotan R, Mangelsdorf DJ, Gronemeyer H (2006). International Union of Pharmacology. LXIII. Retinoid X Receptors. Pharmacol Rev.

[10] Weikum ER, Liu X, Ortlund EA (2018). The Nuclear Receptor Superfamily: A Structural Perspective. Protein Sci.

[11] Kumar R, Thompson EB (2003). Transactivation Functions of the N-Terminal Domains of Nuclear Hormone Receptors: Protein Folding and Coactivator Interactions. Molecular Endocrinology.

[12] Anbalagan M, Huderson B, Murphy L, Rowan BG (2012). Post-Translational Modifications of Nuclear Receptors and Human Disease. Nucl Recept Signal.

[13] Evans RM (1988). The Steroid and Thyroid Hormone Receptor Superfamily. Science (1979).

[14] Helsen C, Kerkhofs S, Clinckemalie L, Spans L, Laurent M, Boonen S, Vanderschueren D, Claessens F (2012). Structural Basis for Nuclear Hormone Receptor DNA Binding. Mol Cell Endocrinol.

[15] Watson LC, Kuchenbecker KM, Schiller BJ, Gross JD, Pufall MA, Yamamoto KR (2013). The Glucocorticoid Receptor Dimer Interface Allosterically Transmits Sequence-Specific DNA Signals. Nat Struct Mol Biol.

[16] Luisi BF, Xu WX, Otwinowski Z, Freedman LP, Yamamoto KR, Sigler PB (1991). Crystallographic Analysis of the Interaction of the Glucocorticoid Receptor with DNA. Nature.

[17] Haelens A, Tanner T, Denayer S, Callewaert L, Claessens F (2007). The Hinge Region Regulates DNA Binding, Nuclear Translocation, and Transactivation of the Androgen Receptor. Cancer Res.

[18] Moras D, Gronemeyer H (1998). The Nuclear Receptor Ligand-Binding Domain: Structure and Function. Curr Opin Cell Biol.

[19] Weatherman RV, Fletterick RJ, Scanlan TS (1999). Nuclear-Receptor Ligands and Ligand-Binding Domains. Annu Rev Biochem.

[20] Bain DL, Heneghan AF, Connaghan-Jones KD, Miura MT (2007). Nuclear Receptor Structure: Implications for Function. Annu Rev Physiol.

[21] Wurtz JM, Bourguet W, Renaud JP, Vivat V, Chambon P, Moras D, Gronemeyer H (1996). A Canonical Structure for the Ligand-Binding Domain of Nuclear Receptors. Nat Struct Biol.

[22] Rastinejad F, Huang P, Chandra V, Khorasanizadeh S (2013). Understanding Nuclear Receptor Form and Function Using Structural Biology. J Mol Endocrinol.

[23] Isigkeit L, Merk D (2023). Opportunities and Challenges in Targeting Orphan Nuclear Receptors. Chemical Communications.

[24] Lewandowski M, Carmina M, Knümann L, Sai M, Willems S, Kasch T, Pollinger J, Knapp S, Marschner JA, Chaikuad A, Merk D (2024). Structure-Guided Design of a Highly Potent Partial RXR Agonist with Superior Physicochemical Properties. J Med Chem.

[25] Díaz-Holguín A, Rashidian A, Pijnenburg D, Monteiro Ferreira G, Stefela A, Kaspar M, Kudova E, Poso A, van Beuningen R, Pavek P, Kronenberger T (2023). When Two Become One: Conformational Changes in FXR/RXR Heterodimers Bound to Steroidal Antagonists. ChemMedChem.

[26] Wang Z, Benoit G, Liu J, Prasad S, Aarnisalo P, Liu X, Xu H, Walker NP, Perlmann T (2003). Structure and Function of Nurr1 Identifies a Class of Ligand-Independent Nuclear Receptors. Nature.

[27] Mangelsdorf DJ, Thummel C, Beato M, Herrlich P, Schütz G, Umesono K, Blumberg B, Kastner P, Mark M, Chambon P, Evans RM (1995). The Nuclear Receptor Superfamily: The Second Decade. Cell.

[28] Shulman AI, Larson C, Mangelsdorf DJ, Ranganathan R (2004). Structural Determinants of Allosteric Ligand Activation in RXR Heterodimers. Cell.

[29] Mangelsdorf DJ, Evans RM (1995). The RXR Heterodimers and Orphan Receptors. Cell.

[30] Minucci S, Leid M, Toyama R, Saint-Jeannet JP, Peterson VJ, Horn V, Ishmael JE, Bhattacharyya N, Dey A, Dawid IB, Ozato K (1997). Retinoid X Receptor (RXR) within the RXR-Retinoic Acid Receptor Heterodimer Binds Its Ligand and Enhances Retinoid-Dependent Gene Expression. Mol Cell Biol.

[31] Zhi X, Zhou XE, He Y, Searose-Xu K, Zhang CL, Tsai CC, Melcher K, Xu HE (2015). Structural Basis for Corepressor Assembly by the Orphan Nuclear Receptor TLX. Genes Dev.

[32] Kobayashi M, Takezawa S, Hara K, Yu RT, Umesono Y, Agata K, Taniwaki M, Yasuda K, Umesono K (1999). Identification of a Photoreceptor Cell-Specific Nuclear Receptor. Proceedings of the National Academy of Sciences.

[33] Xu HE, Stanley TB, Montana VG, Lambert MH, Shearer BG, Cobb JE, McKee DD, Galardi CM, Plunket KD, Nolte RT, Parks DJ (2002). Structural Basis for Antagonist-Mediated Recruitment of Nuclear Co-Repressors by PPARα. Nature.

[34] Gampe RT, Montana VG, Lambert MH, Miller AB, Bledsoe RK, Milburn MV, Kliewer SA, Willson TM, Xu HE (2000). Asymmetry in the PPARγ/RXRα Crystal Structure Reveals the Molecular Basis of Heterodimerization among Nuclear Receptors. Mol Cell.

[35] Jiang S, Tanaka T, Iwanari H, Hotta H, Yamashita H, Kumakura J, Watanabe Y, Uchiyama Y, Aburatani H, Hamakubo T, Kodama T (2003). Expression and Localization of P1 Promoter-Driven Hepatocyte Nuclear Factor-4α (HNF4α) Isoforms in Human and Rats. Nucl Recept.

[36] Vemuri K, Radi SH, Sladek FM, Verzi MP (2023). Multiple Roles and Regulatory Mechanisms of the Transcription Factor HNF4 in the Intestine. Front Endocrinol (Lausanne).

[37] Chandra V, Huang P, Potluri N, Wu D, Kim Y, Rastinejad F (2013). Multidomain Integration in the Structure of the HNF-4α Nuclear Receptor Complex. Nature.

[38] Odom DT, Zizlsperger N, Gordon DB, Bell GW, Rinaldi NJ, Murray HL, Volkert TL, Schreiber J, Rolfe PA, Gifford DK, Fraenkel E (2004). Control of Pancreas and Liver Gene Expression by HNF Transcription Factors. Science (1979).

[39] de Boussac H, Ratajewski M, Sachrajda I, Köblös G, Tordai A, Pulaski L, Buday L, Váradi A, Arányi T (2010). The ERK1/2-Hepatocyte Nuclear Factor 4alpha Axis Regulates Human ABCC6 Gene Expression in Hepatocytes. J Biol Chem.

[40] Hayhurst GP, Lee YH, Lambert G, Ward JM, Gonzalez FJ (2001). Hepatocyte Nuclear Factor 4alpha (Nuclear Receptor 2A1) Is Essential for Maintenance of Hepatic Gene Expression and Lipid Homeostasis. Mol Cell Biol.

[41] Kwon SM, Jung YY, Hwang CJ, Park MH, Yoon NY, Kim TM, Yu JM, Kim DH, Seo DW, Youn HS, Seo HO (2016). Anti-Cancer Effect of N-(3,5-Bis(Trifluoromethyl)Phenyl)-5-Chloro-2,3-Dihydronaphtho[1,2-b]Furan-2-Carboxamide, a Novel Synthetic Compound. Mol Carcinog.

[42] Colclough K, Bellanne-Chantelot C, Saint-Martin C, Flanagan SE, Ellard S (2013). Mutations in the Genes Encoding the Transcription Factors Hepatocyte Nuclear Factor 1 Alpha and 4 Alpha in Maturity-Onset Diabetes of the Young and Hyperinsulinemic Hypoglycemia. Hum Mutat.

[43] Stoffel M, Duncan SA (1997). The Maturity-Onset Diabetes of the Young (MODY1) Transcription Factor HNF4α Regulates Expression of Genes Required for Glucose Transport and Metabolism. Proceedings of the National Academy of Sciences.

[44] Yang Y, Zhou T-C, Liu Y-Y, Li X, Wang W-X, Irwin DM, Zhang Y-P (2016). Identification of HNF4A Mutation p.T130I and HNF1A Mutations p.I27L and p.S487N in a Han Chinese Family with Early-Onset Maternally Inherited Type 2 Diabetes. J Diabetes Res.

[45] Sharma S, Shen T, Chitranshi N, Gupta V, Basavarajappa D, Sarkar S, Mirzaei M, You Y, Krezel W, Graham SL, Gupta V (2022). Retinoid X Receptor: Cellular and Biochemical Roles of Nuclear Receptor with a Focus on Neuropathological Involvement. Mol Neurobiol.

[46] Schierle S, Merk D (2019). Therapeutic Modulation of Retinoid X Receptors – SAR and Therapeutic Potential of RXR Ligands and Recent Patents. Expert Opin Ther Pat.

[47] Boehm MF, Zhang L, Zhi L, McClurg MR, Berger E, Wagoner M, Mais DE, Suto CM, Davies PJA, Heyman RA, Nadzan AM (1995). Design and Synthesis of Potent Retinoid X Receptor Selective Ligands That Induce Apoptosis in Leukemia Cells. J Med Chem.

[48] Altucci L, Leibowitz MD, Ogilvie KM, de Lera AR, Gronemeyer H (2007). RAR and RXR Modulation in Cancer and Metabolic Disease. Nat Rev Drug Discov.

[49] Qi L, Guo Y, Zhang P, Cao X, Luan Y (2016). Preventive and Therapeutic Effects of the Retinoid X Receptor Agonist Bexarotene on Tumors. Curr Drug Metab.

[50] Shen D, Yu X, Wu Y, Chen Y, Li G, Cheng F, Xia L (2018). Emerging Roles of Bexarotene in the Prevention, Treatment and Anti-Drug Resistance of Cancers. Expert Rev Anticancer Ther.

[51] Huang JK, Jarjour AA, Nait Oumesmar B, Kerninon C, Williams A, Krezel W, Kagechika H, Bauer J, Zhao C, Baron-Van Evercooren A, Chambon P (2011). Retinoid X Receptor Gamma Signaling Accelerates CNS Remyelination. Nat Neurosci.

[52] Chandraratna RA, Noelle RJ, Nowak EC (2016). Treatment with Retinoid X Receptor Agonist IRX4204 Ameliorates Experimental Autoimmune Encephalomyelitis. Am J Transl Res.

[53] Ardura-Fabregat A, Boddeke E, Boza-Serrano A, Brioschi S, Castro-Gomez S, Ceyzériat K, Dansokho C, Dierkes T, Gelders G, Heneka MT, Hoeijmakers L (2017). Targeting Neuroinflammation to Treat Alzheimer’s Disease. CNS Drugs.

[54] Koster KP, Smith C, Valencia-Olvera AC, Thatcher GR, Tai LM, LaDu MJ (2017). Rexinoids as Therapeutics for Alzheimer’s Disease: Role of APOE. Curr Top Med Chem.

[55] Cramer PE, Cirrito JR, Wesson DW, Lee CY, Karlo JC, Zinn AE, Casali BT, Restivo JL, Goebel WD, James MJ, Brunden KR (2012). ApoE-Directed Therapeutics Rapidly Clear β-Amyloid and Reverse Deficits in AD Mouse Models. Science (1979).

[56] Cummings JL, Zhong K, Kinney JW, Heaney C, Moll-Tudla J, Joshi A, Pontecorvo M, Devous M, Tang A, Bena J (2016). Double-Blind, Placebo-Controlled, Proof-of-Concept Trial of Bexarotene in Moderate Alzheimer’s Disease. Alzheimers Res Ther.

[57] Willems S, Zaienne D, Merk D (2021). Targeting Nuclear Receptors in Neurodegeneration and Neuroinflammation. J Med Chem.

[58] Kim E, Liu NC, Yu IC, Lin HY, Lee YF, Sparks JD, Chen LM, Chang C (2011). Metformin Inhibits Nuclear Receptor TR4-Mediated Hepatic Stearoyl-CoA Desaturase 1 Gene Expression with Altered Insulin Sensitivity. Diabetes.

[59] Liu NC, Lin WJ, Kim E, Collins LL, Lin HY, Yu IC, Sparks JD, Chen LM, Lee YF, Chang C (2007). Loss of TR4 Orphan Nuclear Receptor Reduces Phosphoenolpyruvate Carboxykinase-Mediated Gluconeogenesis. Diabetes.

[60] Lin S-J, Ho H-C, Lee Y-F, Liu N-C, Liu S, Li G, Shyr C-R, Chang C (2012). Reduced Osteoblast Activity in the Mice Lacking TR4 Nuclear Receptor Leads to Osteoporosis. Reproductive Biology and Endocrinology.

[61] Chen Y-T, Collins LL, Uno H, Chou SM, Meshul CK, Chang S-S, Chang C (2007). Abnormal Cerebellar Cytoarchitecture and Impaired Inhibitory Signaling in Adult Mice Lacking TR4 Orphan Nuclear Receptor. Brain Res.

[62] Chen YT, Collins LL, Uno H, Chang C (2005). Deficits in Motor Coordination with Aberrant Cerebellar Development in Mice Lacking Testicular Orphan Nuclear Receptor 4. Mol Cell Biol.

[63] Bookout AL, Jeong Y, Downes M, Yu RT, Evans RM, Mangelsdorf DJ (2006). Anatomical Profiling of Nuclear Receptor Expression Reveals a Hierarchical Transcriptional Network. Cell.

[64] Lee YF, Lee HJ, Chang C (2002). Recent Advances in the TR2 and TR4 Orphan Receptors of the Nuclear Receptor Superfamily. J Steroid Biochem Mol Biol.

[65] Ding X, Yang D-R, Lee SO, Chen Y-L, Xia L, Lin S-J, Yu S, Niu Y-J, Li G, Chang C (2015). TR4 Nuclear Receptor Promotes Prostate Cancer Metastasis via Upregulation of CCL2/CCR2 Signaling. Int J Cancer.

[66] Shyr CR, Hu YC, Kim E, Chang C (2002). Modulation of Estrogen Receptor-Mediated Transactivation by Orphan Receptor TR4 in MCF-7 Cells. J Biol Chem.

[67] Du L, Bergsneider M, Mirsadraei L, Young SH, Jonker JW, Downes M, Yong WH, Evans RM, Heaney AP (2013). Evidence for Orphan Nuclear Receptor TR4 in the Etiology of Cushing Disease. Proc Natl Acad Sci U S A.

[68] Lin SJ, Yang DR, Yang G, Lin CY, Chang HC, Li G, Chang C (2017). TR2 and TR4 Orphan Nuclear Receptors: An Overview. Curr Top Dev Biol.

[69] Uhlén M, Fagerberg L, Hallström BM, Lindskog C, Oksvold P, Mardinoglu A, Sivertsson Å, Kampf C, Sjöstedt E, Asplund A, Olsson I (2015). Tissue-Based Map of the Human Proteome. Science (1979).

[70] Wang D, Eraslan B, Wieland T, Hallström B, Hopf T, Zolg DP, Zecha J, Asplund A, Li L, Meng C, Frejno M (2019). A Deep Proteome and Transcriptome Abundance Atlas of 29 Healthy Human Tissues. Mol Syst Biol.

[71] Islam MM, Zhang CL (2015). TLX: A Master Regulator for Neural Stem Cell Maintenance and Neurogenesis. Biochim Biophys Acta.

[72] Murai K, Qu Q, Sun G, Ye P, Li W, Asuelime G, Sun E, Tsai GE, Shi Y (2014). Nuclear Receptor TLX Stimulates Hippocampal Neurogenesis and Enhances Learning and Memory in a Transgenic Mouse Model. Proc Natl Acad Sci U S A.

[73] Nelson AT, Wang Y, Nelson ER (2021). TLX, an Orphan Nuclear Receptor With Emerging Roles in Physiology and Disease. Endocrinology.

[74] Liu HK, Belz T, Bock D, Takacs A, Wu H, Lichter P, Chai M, Schütz G (2008). The Nuclear Receptor Tailless Is Required for Neurogenesis in the Adult Subventricular Zone. Genes Dev.

[75] Monaghan AP, Bock D, Gass P, Schwäger A, Wolfer DP, Lipp HP, Schütz G (1997). Defective Limbic System in Mice Lacking the Tailless Gene. Nature.

[76] Kumar RA, McGhee KA, Leach S, Bonaguro R, Maclean A, Aguirre-Hernandez R, Abrahams BS, Coccaro EF, Hodgins S, Turecki G, Condon A (2008). Initial Association of NR2E1 with Bipolar Disorder and Identification of Candidate Mutations in Bipolar Disorder, Schizophrenia, and Aggression through Resequencing. Am J Med Genet B Neuropsychiatr Genet.

[77] Murai K, Sun G, Ye P, Tian E, Yang S, Cui Q, Sun G, Trinh D, Sun O, Hong T, Wen Z (2016). The TLX-MiR-219 Cascade Regulates Neural Stem Cell Proliferation in Neurodevelopment and Schizophrenia IPSC Model. Nat Commun.

[78] Chen J, Rattner A, Nathans J (2005). The Rod Photoreceptor-Specific Nuclear Receptor Nr2e3 Represses Transcription of Multiple Cone-Specific Genes. J Neurosci.

[79] Cheng H, Khan NW, Roger JE, Swaroop A (2011). Excess Cones in the Retinal Degeneration Rd7 Mouse, Caused by the Loss of Function of Orphan Nuclear Receptor Nr2e3, Originate from Early-Born Photoreceptor Precursors. Hum Mol Genet.

[80] Haider NB, Demarco P, Nystuen AM, Huang X, Smith RS, McCall MA, Naggert JK, Nishina PM (2006). The Transcription Factor Nr2e3 Functions in Retinal Progenitors to Suppress Cone Cell Generation. Vis Neurosci.

[81] Haider NB, Naggert JK, Nishina PM (2001). Excess Cone Cell Proliferation Due to Lack of a Functional NR2E3 Causes Retinal Dysplasia and Degeneration in Rd7/Rd7 Mice. Hum Mol Genet.

[82] Olivares AM, Jelcick AS, Reinecke J, Leehy B, Haider A, Morrison MA, Cheng L, Chen DF, DeAngelis MM, Haider NB (2017). Multimodal Regulation Orchestrates Normal and Complex Disease States in the Retina. Sci Rep.

[83] Cheng H, Khanna H, Oh EC, Hicks D, Mitton KP, Swaroop A (2004). Photoreceptor-Specific Nuclear Receptor NR2E3 Functions as a Transcriptional Activator in Rod Photoreceptors. Hum Mol Genet.

[84] Gerber S, Rozet JM, Takezawa SI, dos Santos LC, Lopes L, Gribouval O, Penet C, Perrault I, Ducroq D, Souied E, Jeanpierre M (2000). The Photoreceptor Cell-Specific Nuclear Receptor Gene (PNR) Accounts for Retinitis Pigmentosa in the Crypto-Jews from Portugal (Marranos), Survivors from the Spanish Inquisition. Hum Genet.

[85] Haider NB, Jacobson SG, Cideciyan AV, Swiderski R, Streb LM, Searby C, Beck G, Hockey R, Hanna DB, Gorman S, Duhl D (2000). Mutation of a Nuclear Receptor Gene, NR2E3, Causes Enhanced S Cone Syndrome, a Disorder of Retinal Cell Fate. Nat Genet.

[86] Sharon D, Sandberg MA, Caruso RC, Berson EL, Dryja TP (2003). Shared Mutations in NR2E3 in Enhanced S-Cone Syndrome, Goldmann-Favre Syndrome, and Many Cases of Clumped Pigmentary Retinal Degeneration. Arch Ophthalmol.

[87] Tsai SY, Tsai M-J (1997). Chick Ovalbumin Upstream Promoter-Transcription Factors (COUP-TFs): Coming of Age. Endocr Rev.

[88] Pereira FA, Tsai MJ, Tsai SY (2000). COUP-TF Orphan Nuclear Receptors in Development and Differentiation. Cell Mol Life Sci.

[89] Yu CT, Tang K, Suh JM, Jiang R, Tsai SY, Tsai MJ (2012). COUP-TFII Is Essential for Metanephric Mesenchyme Formation and Kidney Precursor Cell Survival. Development.

[90] Xie X, Qin J, Lin SH, Tsai SY, Tsai MJ (2011). Nuclear Receptor Chicken Ovalbumin Upstream Promoter-Transcription Factor II (COUP-TFII) Modulates Mesenchymal Cell Commitment and Differentiation. Proc Natl Acad Sci U S A.

[91] Pereira FA, Qiu Y, Zhou G, Tsai MJ, Tsai SY (1999). The Orphan Nuclear Receptor COUP-TFII Is Required for Angiogenesis and Heart Development. Genes Dev.

[92] Qin J, Chen X, Xie X, Tsai MJ, Tsai SY (2010). COUP-TFII Regulates Tumor Growth and Metastasis by Modulating Tumor Angiogenesis. Proc Natl Acad Sci U S A.

[93] Niu C, Sun X, Zhang W, Li H, Xu L, Li J, Xu B, Zhang Y (2016). NR2F6 Expression Correlates with Pelvic Lymph Node Metastasis and Poor Prognosis in Early-Stage Cervical Cancer. Int J Mol Sci.

[94] Li XB, Jiao S, Sun H, Xue J, Zhao WT, Fan L, Wu GH, Fang J (2011). The Orphan Nuclear Receptor EAR2 Is Overexpressed in Colorectal Cancer and It Regulates Survivability of Colon Cancer Cells. Cancer Lett.

[95] Klapper L, Ribbat-Idel J, Kuppler P, Paulsen F-O, Bruchhage K-L, Rades D, Offermann A, Kirfel J, Wollenberg B, Idel C, Perner S (2020). NR2F6 as a Prognostic Biomarker in HNSCC. Int J Mol Sci.

[96] Ichim CV, Dervović DD, Zúñiga-Pflücker JC, Wells RA (2014). The Orphan Nuclear Receptor Ear-2 (Nr2f6) Is a Novel Negative Regulator of T Cell Development. Exp Hematol.

[97] Sajinovic T, Baier G (2023). New Insights into the Diverse Functions of the NR2F Nuclear Orphan Receptor Family. Frontiers in Bioscience-Landmark.

[98] Tan MHE, Zhou XE, Soon F-F, Li X, Li J, Yong E-L, Melcher K, Xu HE (2013). The Crystal Structure of the Orphan Nuclear Receptor NR2E3/PNR Ligand Binding Domain Reveals a Dimeric Auto-Repressed Conformation. PLoS One.

[99] Duda K, Chi Y-I, Shoelson SE (2004). Structural Basis for HNF-4α Activation by Ligand and Coactivator Binding. Journal of Biological Chemistry.

[100] Dhe-Paganon S, Duda K, Iwamoto M, Chi Y-I, Shoelson SE (2002). Crystal Structure of the HNF4α Ligand Binding Domain in Complex with Endogenous Fatty Acid Ligand. Journal of Biological Chemistry.

[101] Holzer G, Markov GV, Laudet V (2017). Evolution of Nuclear Receptors and Ligand Signaling: Toward a Soft Key-Lock Model?. Curr Top Dev Biol.

[102] Faudone G, Zhubi R, Celik F, Knapp S, Chaikuad A, Heering J, Merk D (2022). Design of a Potent TLX Agonist by Rational Fragment Fusion. J Med Chem.

[103] Faudone G, Kilu W, Ni X, Chaikuad A, Sreeramulu S, Heitel P, Schwalbe H, Knapp S, Schubert-Zsilavecz M, Heering J, Merk D (2021). The Transcriptional Repressor Orphan Nuclear Receptor TLX Is Responsive to Xanthines. ACS Pharmacol Transl Sci.

[104] Faudone G, Bischoff-Kont I, Rachor L, Willems S, Zhubi R, Kaiser A, Chaikuad A, Knapp S, Fürst R, Heering J, Merk D (2021). Propranolol Activates the Orphan Nuclear Receptor TLX to Counteract Proliferation and Migration of Glioblastoma Cells. J Med Chem.

[105] Dueva E, Singh K, Kalyta A, LeBlanc E, Rennie PS, Cherkasov A (2018). Computer-Aided Discovery of Small Molecule Inhibitors of Transcriptional Activity of TLX (NR2E1) Nuclear Receptor. Molecules.

[106] Gronemeyer H, Gustafsson J-Å, Laudet V (2004). Principles for Modulation of the Nuclear Receptor Superfamily. Nat Rev Drug Discov.

[107] Wisely GB, Miller AB, Davis RG, Johnson R, Spitzer T, Sefler A, Shearer B, Moore JT, Miller AB, Willson TM, Williams SP (2002). Hepatocyte Nuclear Factor 4 Is a Transcription Factor That Constitutively Binds Fatty Acids. Structure.

[108] Lee S-H, Athavankar S, Cohen T, Piran R, Kiselyuk A, Levine F (2013). Identification of Alverine and Benfluorex as HNF4α Activators. ACS Chem Biol.

[109] Kiselyuk A, Lee S-H, Farber-Katz S, Zhang M, Athavankar S, Cohen T, Pinkerton AB, Ye M, Bushway P, Richardson AD, Hostetler HA (2012). HNF4α Antagonists Discovered by a High-Throughput Screen for Modulators of the Human Insulin Promoter. Chem Biol.

[110] Meijer I, Willems S, Ni X, Heering J, Chaikuad A, Merk D (2020). Chemical Starting Matter for HNF4α Ligand Discovery and Chemogenomics. Int J Mol Sci.

[111] de Lera ÁR, Krezel W, Rühl R (2016). An Endogenous Mammalian Retinoid X Receptor Ligand, At Last!. ChemMedChem.

[112] Medvedev A, Moeser M, Medvedeva L, Martsen E, Granick A, Raines L, Gorman K, Lin B, Zeng M, Houck KA, Makarov SS (2022). Comprehensive Assessment of NR Ligand Polypharmacology by a Multiplex Reporter NR Assay. Sci Rep.

[113] Farol LT, Hymes KB (2004). Bexarotene: A Clinical Review. Expert Rev Anticancer Ther.

[114] Lowe MN, Plosker GL (2000). Bexarotene. Am J Clin Dermatol.

[115] Boehm MF, Zhang L, Zhi L, McClurg MR, Berger E, Wagoner M, Mais DE, Suto CM, Davies PJA, Heyman RA, Nadzan AM (1995). Design and Synthesis of Potent Retinoid X Receptor Selective Ligands That Induce Apoptosis in Leukemia Cells. J Med Chem.

[116] Liu Y, Wang P, Jin G, Shi P, Zhao Y, Guo J, Yin Y, Shao Q, Li P, Yang P (2023). The Novel Function of Bexarotene for Neurological Diseases. Ageing Res Rev.

[117] Pollinger J, Gellrich L, Schierle S, Kilu W, Schmidt J, Kalinowsky L, Ohrndorf J, Kaiser A, Heering J, Proschak E, Merk D (2019). Tuning Nuclear Receptor Selectivity of Wy14,643 towards Selective Retinoid X Receptor Modulation. J Med Chem.

[118] Heitel P, Achenbach J, Moser D, Proschak E, Merk D (2017). DrugBank Screening Revealed Alitretinoin and Bexarotene as Liver X Receptor Modulators. Bioorg Med Chem Lett.

[119] Atigadda VR, Vines KK, Grubbs CJ, Hill DL, Beenken SL, Bland KI, Brouillette WJ, Muccio DD (2003). Conformationally Defined Retinoic Acid Analogues. 5. Large-Scale Synthesis and Mammary Cancer Chemopreventive Activity for (2 E, 4 E, 6 Z, 8 E)-8-(3’,4’-Dihydro-1’(2’ H)-Naphthalen-1’-Ylidene)-3,7-Dimethyl-2,4,6-Octatrienoic Acid (9cUAB30). J Med Chem.

[120] Kolesar JM, Andrews S, Green H, Havighurst TC, Wollmer BW, DeShong K, Laux DE, Krontiras H, Muccio DD, Kim K, Grubbs CJ (2019). A Randomized, Placebo-Controlled, Double-Blind, Dose Escalation, Single Dose, and Steady-State Pharmacokinetic Study of 9cUAB30 in Healthy Volunteers. Cancer Prevention Research.

[121] Reich LA, Moerland JA, Leal AS, Zhang D, Carapellucci S, Lockwood B, Jurutka PW, Marshall PA, Wagner CE, Liby KT (2022). The Rexinoid V-125 Reduces Tumor Growth in Preclinical Models of Breast and Lung Cancer. Sci Rep.

[122] Lowenthal J, Hull SC, Pearson SD (2012). The Ethics of Early Evidence — Preparing for a Possible Breakthrough in Alzheimer’s Disease. New England Journal of Medicine.

[123] Adouvi G, Nawa F, Ballarotto M, Rüger LA, Knümann L, Kasch T, Arifi S, Schubert-Zsilavecz M, Willems S, Marschner JA, Pabel J (2023). Structural Fusion of Natural and Synthetic Ligand Features Boosts RXR Agonist Potency. J Med Chem.

[124] Hartung IV, Rudolph J, Mader MM, Mulder MPC, Workman P (2023). Expanding Chemical Probe Space: Quality Criteria for Covalent and Degrader Probes. J Med Chem.

[125] Watanabe M, Kakuta H (2018). Retinoid X Receptor Antagonists. Int J Mol Sci.

[126] Kakuta H, Yakushiji N, Shinozaki R, Ohsawa F, Yamada S, Ohta Y, Kawata K, Nakayama M, Hagaya M, Fujiwara C, Makishima M (2012). RXR Partial Agonist CBt-PMN Exerts Therapeutic Effects on Type 2 Diabetes without the Side Effects of RXR Full Agonists. ACS Med Chem Lett.

[127] Kawata K, Morishita K, Nakayama M, Yamada S, Kobayashi T, Furusawa Y, Arimoto-Kobayashi S, Oohashi T, Makishima M, Naitou H, Ishitsubo E (2015). RXR Partial Agonist Produced by Side Chain Repositioning of Alkoxy RXR Full Agonist Retains Antitype 2 Diabetes Activity without the Adverse Effects. J Med Chem.

[128] Morichika D, Miyahara N, Fujii U, Taniguchi A, Oda N, Senoo S, Kataoka M, Tanimoto M, Kakuta H, Kiura K, Maeda Y (2019). A Retinoid X Receptor Partial Agonist Attenuates Pulmonary Emphysema and Airway Inflammation. Respir Res.

[129] de Lera AR, Bourguet W, Altucci L, Gronemeyer H (2007). Design of Selective Nuclear Receptor Modulators: RAR and RXR as a Case Study. Nat Rev Drug Discov.

[130] Chaikuad A, Pollinger J, Rühl M, Ni X, Kilu W, Heering J, Merk D (2020). Comprehensive Set of Tertiary Complex Structures and Palmitic Acid Binding Provide Molecular Insights into Ligand Design for RXR Isoforms. Int J Mol Sci.

[131] Merk D, Grisoni F, Friedrich L, Gelzinyte E, Schneider G (2018). Computer-Assisted Discovery of Retinoid X Receptor Modulating Natural Products and Isofunctional Mimetics. J Med Chem.

[132] Pollinger J, Schierle S, Gellrich L, Ohrndorf J, Kaiser A, Heitel P, Chaikuad A, Knapp S, Merk D (2019). A Novel Biphenyl-Based Chemotype of Retinoid X Receptor Ligands Enables Subtype and Heterodimer Preferences. ACS Med Chem Lett.

[133] Adouvi G, Isigkeit L, López-García Ú, Chaikuad A, Marschner JA, Schubert-Zsilavecz M, Merk D (2023). Rational Design of a New RXR Agonist Scaffold Enabling Single-Subtype Preference for RXRα, RXRβ, and RXRγ. J Med Chem.

[134] Takamatsu K, Takano A, Yakushiji N, Morohashi K, Morishita K, Matsuura N, Makishima M, Tai A, Sasaki K, Kakuta H (2008). The First Potent Subtype-Selective Retinoid X Receptor (RXR) Agonist Possessing a 3-Isopropoxy-4-Isopropylphenylamino Moiety, NEt-3IP (RXRα/β-Dual Agonist). ChemMedChem.

[135] Takamatsu K, Takano A, Yakushiji N, Morishita K, Matsuura N, Makishima M, Ali HI, Akaho E, Tai A, Sasaki K, Kakuta H (2008). Reduction of Lipophilicity at the Lipophilic Domain of RXR Agonists Enables Production of Subtype Preference: RXRα-Preferential Agonist Possessing a Sulfonamide Moiety. ChemMedChem.

[136] Vuligonda V, Thacher SM, Chandraratna RAS (2001). Enantioselective Syntheses of Potent Retinoid X Receptor Ligands: Differential Biological Activities of Individual Antipodes. J Med Chem.

[137] Germain P, Iyer J, Zechel C, Gronemeyer H (2002). Co-Regulator Recruitment and the Mechanism of Retinoic Acid Receptor Synergy. Nature.

[138] Sakaki J, Konishi K, Kishida M, Gunji H, Kanazawa T, Uchiyama H, Fukaya H, Mitani H, Kimura M (2007). Synthesis and Structure–Activity Relationship of RXR Antagonists Based on the Diazepinylbenzoic Acid Structure. Bioorg Med Chem Lett.

[139] Ebisawa M, Umemiya H, Ohta K, Fukasawa H, Kawachi E, Christoffel G, Gronemeyer H, Tsuji M, Hashimoto Y, Shudo K, Kagechika H (1999). Retinoid X Receptor-Antagonistic Diazepinylbenzoic Acids. Chem Pharm Bull (Tokyo).

[140] Solt LA, Wang Y, Banerjee S, Hughes T, Kojetin DJ, Lundasen T, Shin Y, Liu J, Cameron MD, Noel R, Yoo S-H (2012). Regulation of Circadian Behaviour and Metabolism by Synthetic REV-ERB Agonists. Nature.

[141] Benod C, Villagomez R, Filgueira CS, Hwang PK, Leonard PG, Poncet-Montange G, Rajagopalan S, Fletterick RJ, Gustafsson J-Å, Webb P (2014). The Human Orphan Nuclear Receptor Tailless (TLX, NR2E1) Is Druggable. PLoS One.

[142] Griffett K, Bedia-Diaz G, Hegazy L, de Vera IMS, Wanninayake US, Billon C, Koelblen T, Wilhelm ML, Burris TP (2020). The Orphan Nuclear Receptor TLX Is a Receptor for Synthetic and Natural Retinoids. Cell Chem Biol.

[143] Kapitskaya M, Cunningham M, Lacson R, Kornienko O, Bednar B, Petrukhin K (2006). Development of the High Throughput Screening Assay for Identification of Agonists of an Orphan Nuclear Receptor. Assay Drug Dev Technol.

[144] Qiong Q, Knapinska A, Dobri N, Madoux F, Chase P, Hodder P, Petrukhin K (2013). In Pursuit of Synthetic Modulators for the Orphan Retina-Specific Nuclear Receptor NR2E3. Journal of Ocular Pharmacology and Therapeutics.

[145] Nakamura PA, Shimchuk AA, Tang S, Wang Z, DeGolier K, Ding S, Reh TA (2017). Small Molecule Photoregulin3 Prevents Retinal Degeneration in the RhoP23H Mouse Model of Retinitis Pigmentosa. Elife.

[146] Nakamura PA, Tang S, Shimchuk AA, Ding S, Reh TA (2016). Potential of Small Molecule–Mediated Reprogramming of Rod Photoreceptors to Treat Retinitis Pigmentosa. Invest Ophthalmol Vis Sci.

[147] Forrest D, Swaroop A (2012). Minireview: The Role of Nuclear Receptors in Photoreceptor Differentiation and Disease. Mol Endocrinol.

[148] Mollema NJ, Yuan Y, Jelcick AS, Sachs AJ, von Alpen D, Schorderet D, Escher P, Haider NB (2011). Nuclear Receptor Rev-Erb Alpha (Nr1d1) Functions in Concert with Nr2e3 to Regulate Transcriptional Networks in the Retina. PLoS One.

[149] Onishi A, Peng G-H, Poth EM, Lee DA, Chen J, Alexis U, de Melo J, Chen S, Blackshaw S (2010). The Orphan Nuclear Hormone Receptor ERRβ Controls Rod Photoreceptor Survival. Proceedings of the National Academy of Sciences.

[150] Jia L, Oh ECT, Ng L, Srinivas M, Brooks M, Swaroop A, Forrest D (2009). Retinoid-Related Orphan Nuclear Receptor RORβ Is an Early-Acting Factor in Rod Photoreceptor Development. Proceedings of the National Academy of Sciences.

[151] Connor B, Titialii-Torres K, Rockenhaus AE, Passamonte S, Morris AC, Lee YS (2022). Biliverdin Regulates NR2E3 and Zebrafish Retinal Photoreceptor Development. Sci Rep.

[152] Haider NB, Mollema N, Gaule M, Yuan Y, Sachs AJ, Nystuen AM, Naggert JK, Nishina PM (2009). Nr2e3-Directed Transcriptional Regulation of Genes Involved in Photoreceptor Development and Cell-Type Specific Phototransduction. Exp Eye Res.

[153] Khalil BD, Sanchez R, Rahman T, Rodriguez-Tirado C, Moritsch S, Martinez AR, Miles B, Farias E, Mezei M, Nobre AR, Singh D (2021). An NR2F1-Specific Agonist Suppresses Metastasis by Inducing Cancer Cell Dormancy. Journal of Experimental Medicine.

[154] Wang L, Cheng C-M, Qin J, Xu M, Kao C-Y, Shi J, You E, Gong W, Rosa LP, Chase P, Scampavia L (2024). Small-Molecule Inhibitor Targeting Orphan Nuclear Receptor COUP-TFII for Prostate Cancer Treatment. Sci Adv.

[155] Benod C, Villagomez R, Filgueira CS, Hwang PK, Leonard PG, Poncet-Montange G, Rajagopalan S, Fletterick RJ, Gustafsson J-Å, Webb P (2014). The Human Orphan Nuclear Receptor Tailless (TLX, NR2E1) Is Druggable. PLoS One.

[156] Griffett K, Bedia-Diaz G, Hegazy L, de Vera IMS, Wanninayake US, Billon C, Koelblen T, Wilhelm ML, Burris TP (2020). The Orphan Nuclear Receptor TLX Is a Receptor for Synthetic and Natural Retinoids. Cell Chem Biol.

[157] Wolkenberg SE, Zhao Z, Kapitskaya M, Webber AL, Petrukhin K, Tang YS, Dean DC, Hartman GD, Lindsley CW (2006). Identification of Potent Agonists of Photoreceptor-Specific Nuclear Receptor (NR2E3) and Preparation of a Radioligand. Bioorg Med Chem Lett.

[158] Arifi S, Marschner JA, Pollinger J, Isigkeit L, Heitel P, Kaiser A, Obeser L, Höfner G, Proschak E, Knapp S, Chaikuad A (2023). Targeting the Alternative Vitamin E Metabolite Binding Site Enables Noncanonical PPARγ Modulation. J Am Chem Soc.

[159] Heering J, Merk D (2019). Hybrid Reporter Gene Assays: Versatile In Vitro Tools to Characterize Nuclear Receptor Modulators. Methods Mol Biol.

[160] Sadowski I, Ma J, Triezenberg S, Ptashne M (1988). GAL4-VP16 Is an Unusually Potent Transcriptional Activator. Nature.

[161] Isigkeit L, Schallmayer E, Busch R, Brunello L, Menge A, Elson L, Müller S, Knapp S, Stolz A, Marschner JA, Merk D (2024). Chemogenomics for NR1 Nuclear Hormone Receptors. Nat Commun.

[162] Wolbank S, Stadler G, Peterbauer A, Gillich A, Karbiener M, Streubel B, Wieser M, Katinger H, van Griensven M, Redl H, Gabriel C (2009). Telomerase Immortalized Human Amnion-and Adipose-Derived Mesenchymal Stem Cells: Maintenance of Differentiation and Immunomodulatory Characteristics. Tissue Eng Part A.

[163] Cristancho AG, Lazar MA (2011). Forming Functional Fat: A Growing Understanding of Adipocyte Differentiation. Nat Rev Mol Cell Biol.

[164] Chen X, Shen J, Prywes R (2002). The Luminal Domain of ATF6 Senses Endoplasmic Reticulum (ER) Stress and Causes Translocation of ATF6 from the ER to the Golgi. J Biol Chem.

[165] Kim S, Chen J, Cheng T, Gindulyte A, He J, He S, Li Q, Shoemaker BA, Thiessen PA, Yu B, Zaslavsky L (2023). PubChem 2023 Update. Nucleic Acids Res.

[166] Zdrazil B, Felix E, Hunter F, Manners EJ, Blackshaw J, Corbett S, de Veij M, Ioannidis H, Lopez DM, Mosquera JF, Magarinos MP (2024). The ChEMBL Database in 2023: A Drug Discovery Platform Spanning Multiple Bioactivity Data Types and Time Periods. Nucleic Acids Res.

[167] Harding SD, Armstrong JF, Faccenda E, Southan C, Alexander SPH, Davenport AP, Spedding M, Davies JA (2024). The IUPHAR/BPS Guide to PHARMACOLOGY in 2024. Nucleic Acids Res.

[168] Antolin AA, Sanfelice D, Crisp A, Villasclaras Fernandez E, Mica IL, Chen Y, Collins I, Edwards A, Müller S, Al-Lazikani B, Workman P (2023). The Chemical Probes Portal: An Expert Review-Based Public Resource to Empower Chemical Probe Assessment, Selection and Use. Nucleic Acids Res.

[169] Skuta C, Popr M, Muller T, Jindrich J, Kahle M, Sedlak D, Svozil D, Bartunek P (2017). Probes &amp; Drugs Portal: An Interactive, Open Data Resource for Chemical Biology. Nat Methods.

[170] Gellrich L, Heitel P, Heering J, Kilu W, Pollinger J, Goebel T, Kahnt A, Arifi S, Pogoda W, Paulke A, Steinhilber D (2020). L-Thyroxin and the Nonclassical Thyroid Hormone TETRAC Are Potent Activators of PPARγ. J Med Chem.

[171] Flesch D, Cheung S-Y, Schmidt J, Gabler M, Heitel P, Kramer J, Kaiser A, Hartmann M, Lindner M, Lüddens-Dämgen K, Heering J (2017). Nonacidic Farnesoid X Receptor Modulators. J Med Chem.

[172] Rau O, Wurglics M, Paulke A, Zitzkowski J, Meindl N, Bock A, Dingermann T, Abdel-Tawab M, Schubert-Zsilavecz M (2006). Carnosic Acid and Carnosol, Phenolic Diterpene Compounds of the Labiate Herbs Rosemary and Sage, Are Activators of the Human Peroxisome Proliferator-Activated Receptor Gamma. Planta Med.

[173] Moret M, Helmstädter M, Grisoni F, Schneider G, Merk D (2021). Beam Search for Automated Design and Scoring of Novel ROR Ligands with Machine Intelligence**. Angewandte Chemie International Edition.

[174] Schmidt J, Klingler F-M, Proschak E, Steinhilber D, Schubert-Zsilavecz M, Merk D (2015). NSAIDs Ibuprofen, Indometacin and Diclofenac Do Not Interact with Farnesoid X Receptor. Sci Rep.

[175] Heitel P, Gellrich L, Kalinowsky L, Heering J, Kaiser A, Ohrndorf J, Proschak E, Merk D (2019). Computer-Assisted Discovery and Structural Optimization of a Novel Retinoid X Receptor Agonist Chemotype. ACS Med Chem Lett.

[176] Willems S, Kilu W, Ni X, Chaikuad A, Knapp S, Heering J, Merk D (2020). The Orphan Nuclear Receptor Nurr1 Is Responsive to Non-Steroidal Anti-Inflammatory Drugs. Commun Chem.

