# Supplementary material for: Comparative Evaluation and Profiling of Chemical Tools for the Nuclear Hormone Receptor Family 2
Source: ACS Pharmacol Transl Sci. Author manuscript; Available in PMC 2025 Mar 4. (PMC7617459; doi:10.1021/acsptsci.4c00719)
Supplement: Supporting info. [file EMS203540-supplement-Supporting_info_.pdf]

-Supporting Information-

# Comparative Evaluation and Profiling of Chemical Tools for NR2 Nuclear Hormone Receptors

Max Lewandowski[1], Romy Busch[1], Julian A. Marschner[1], Daniel Merk[1]*

[1] Ludwig-Maximilians-Universität München, Department of Pharmacy, 81377 Munich, Germany
* daniel.merk@cup.lmu.de

**Table of Contents**

Figures S1-S4 ........................................................................................................................ S2

# Supplementary Figures

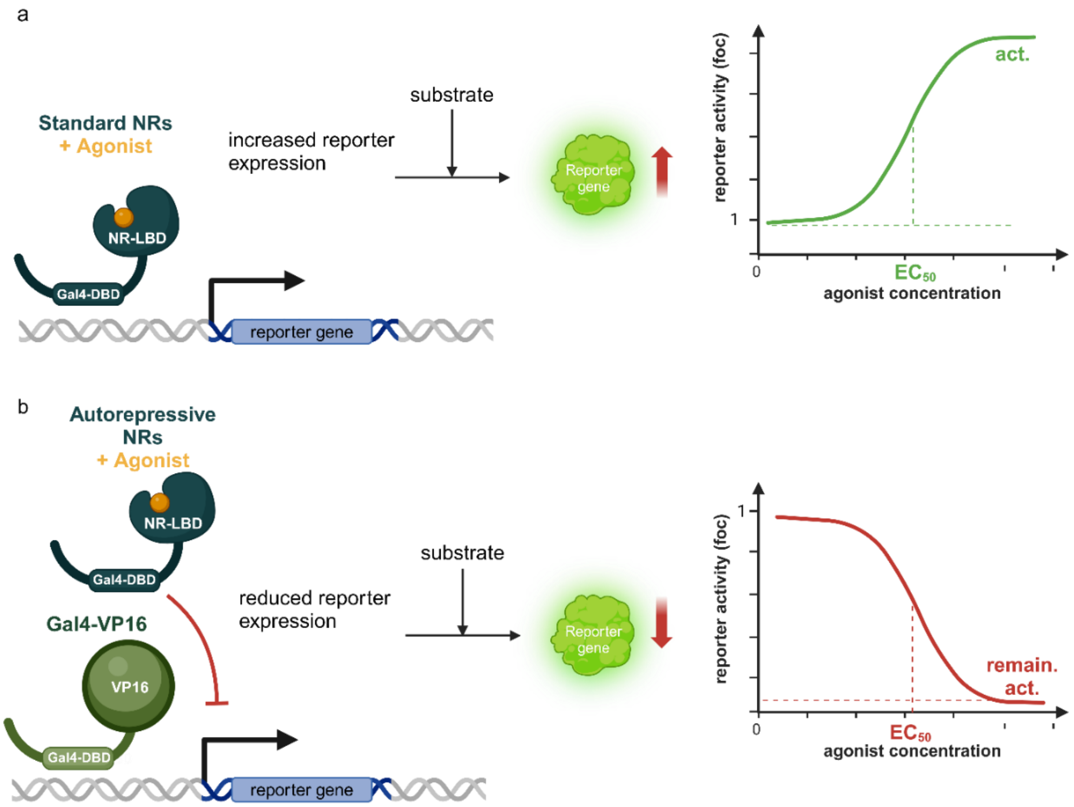


**Figure S1**. **Schematic illustration of Gal4-hybrid reporter gene assays for conventional nuclear receptors (a) and transcriptional repressors (b).** (a) Agonist binding to the Gal4-NR hybrid of a conventional NR (e.g., RXR) induces firefly luciferase reporter gene expression, resulting in increased reporter activity compared to the DMSO control. (b) To capture repressor activity in the Gal4-hybrid system, firefly luciferase reporter gene expression is induced by the transcriptional activator Gal4-VP16. The Gal4-hybrid of an NR acting as transcriptional repressor suppresses the Gal4-VP16 induced reporter expression and agonist binding enhances this repressor activity leading to reduced reporter activity (relative to DMSO control). Efficacy is represented by the remaining reporter activity (% remain. act.). Created in https://BioRender.com.

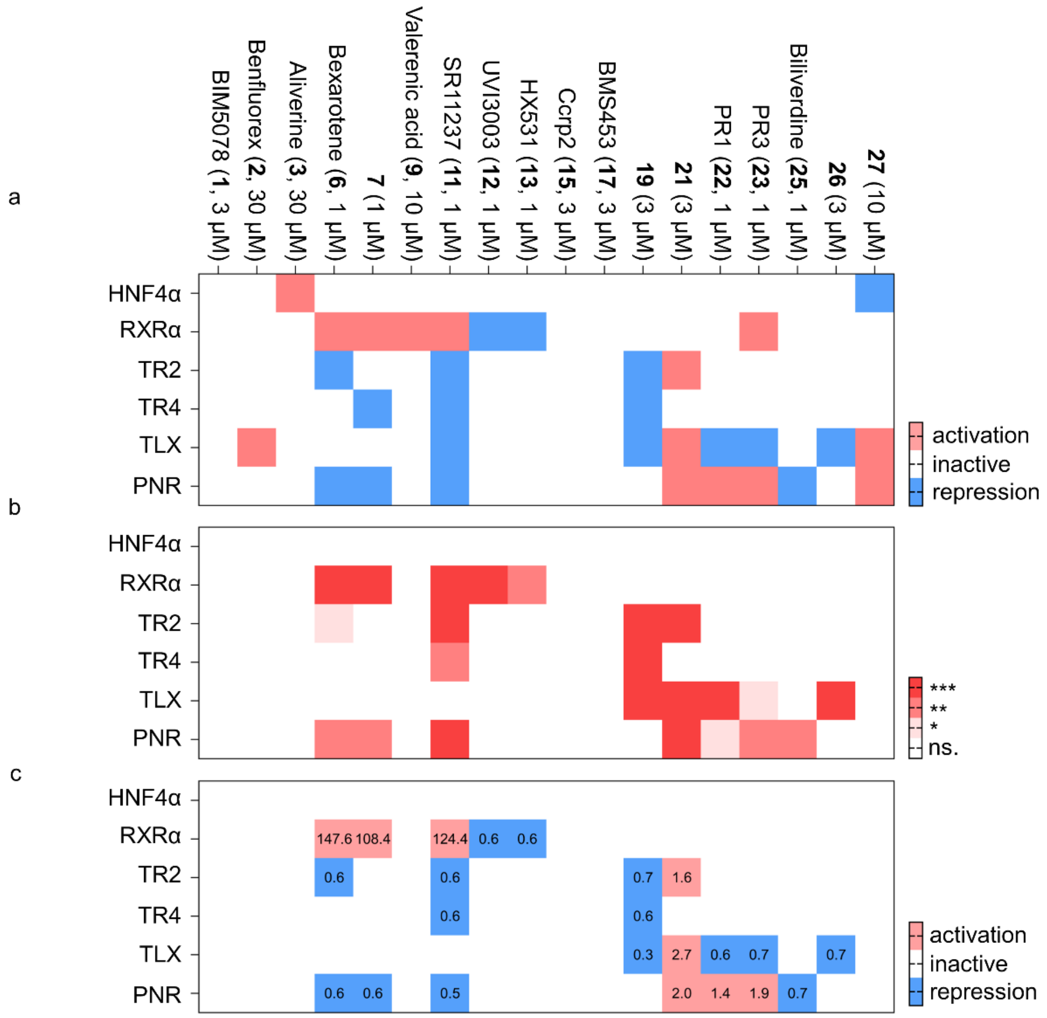


**Figure S2**. In-family profiling of NR2 modulators. (a) The heatmap shows NR activation (red; activation, fA > 1.3) and inhibition (blue, antagonist and inverse agonists, fA < 0.7) by NR2 modulators at the indicated concentrations. All activities were determined in uniform Gal4-hybrid reporter gene assays. (b) Significance level of compound effects on the respective NR target vs. effects on VP16 activity (n.s. – not significant ($p \geq 0.05$), * $p < 0.05$, ** $p < 0.01$; *** $p < 0.001$; two-sided t-test). (c) Activity profiles of NR2 modulators filtered for statistically significant effects vs. VP16 control. All data are n≥3.

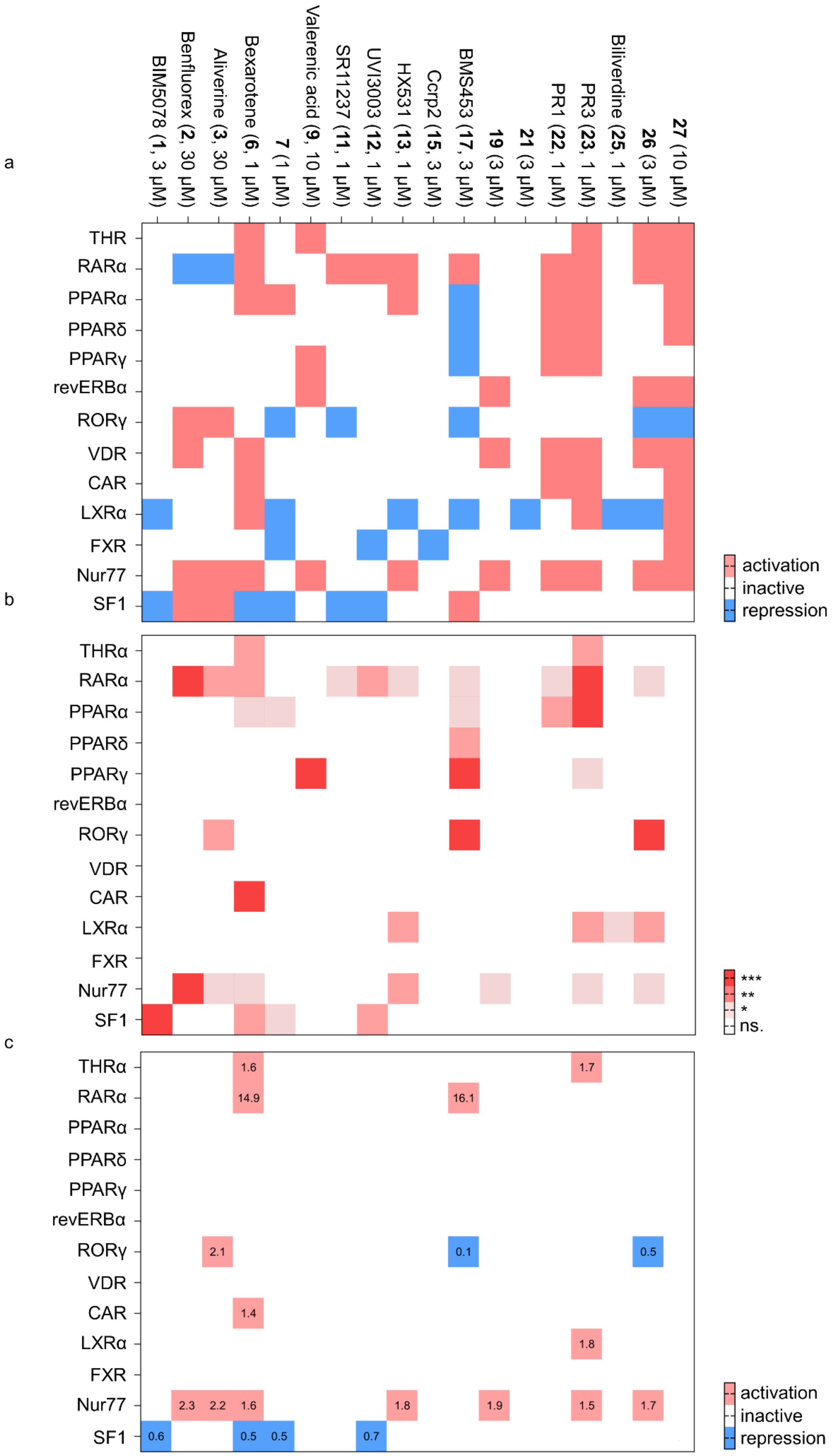


**Figure S3**. Selectivity profiling of NR2 modulators outside the NR2 family. (a) The heatmap shows NR activation (red; activation, fA > 1.3) and inhibition (blue, antagonist and inverse agonists, fA < 0.7) by NR2 modulators at the indicated concentrations. All activities were determined in uniform Gal4-hybrid reporter gene assays. (b) Significance level of compound effects on the respective NR target vs. effects on VP16 activity (n.s. – not significant ($p \geq 0.05$), * $p < 0.05$, ** $p < 0.01$; *** $p < 0.001$; two-sided t-test). (c) Activity profiles of NR2 modulators filtered for statistically significant effects vs. VP16 control. Effects <5% of the respective reference ligands were considered as not relevant and omitted. All data are n≥3.
